# SETD7 promotes LC3B methylation and degradation in ovarian cancer

**DOI:** 10.1016/j.jbc.2024.108134

**Published:** 2024-12-25

**Authors:** Ziwei Zhang, Mingyang Li, Yanan Hou, Ting Huang, Bowen Zhang, Qiong Lin, Genbao Shao

**Affiliations:** Department of Basic Medicine, School of Medicine, Jiangsu University, Zhenjiang, Jiangsu, China

**Keywords:** SETD7, LC3B, autophagy, Posttranslational modification, ovarian cancer

## Abstract

Microtubule-associated protein 1 light chain 3 (LC3) is a key autophagy-related protein involved in regulating autophagosome formation and autophagy activity. Post-translational modifications of LC3 are necessary to modulate its function. However, LC3 protein methylation and its physiological significance have not yet been elucidated. Here, we show that SET domain containing lysine methyltransferase 7 (SETD7) interacts with LC3B, a common isoform of LC3, and methylates LC3B at lysine 51 (K51). SETD7-mediated methylation of LC3B promotes ubiquitination and degradation of LC3B, resulting in reduced autophagosome formation. Furthermore, SETD7 exerts a tumor-promotive function in ovarian cancer (OC) cells in a K51 methylation-dependent manner. Collectively, our data define a novel modification of LC3B and highlight the oncogenic effect of SETD7 *via* mediating LC3B methylation and degradation.

The autophagy protein microtubule-associated protein 1 light chain 3 (LC3) is subject to post-translational modifications (PTMs), which are essential for controlling LC3 activities and the autophagy process (1). The synthesis of LC3 PTMs can contribute to a multilayered post-translational regulatory network (2). Ubiquitylation of LC3B, a common isoform of LC3, at lysine 51 (K51) by the ubiquitin ligase BIRC6 triggers its proteasome-mediated degradation (3). Acetylation at K49 and/or K51 may help stabilize LC3B by preventing ubiquitylation at K51 (4, 5), which otherwise causes proteasome-dependent degradation of LC3B (3). Recent reports have described the role of LC3B phosphorylation in controlling autophagosomal transport (2, 6). The serine/threonine kinase STK4-mediated phosphorylation of LC3B at threonine 50 (T50) is essential for regulating the directional transport of autophagosome (6). However, other types of PTMs, especially methylation, and their roles in modifying LC3B stability and activity remain unclear.

Protein lysine methyltransferases (PKMTs) have been shown to methylate histone and non-histone proteins and participate in regulating several biological processes in both healthy and pathological contexts (7). SET domain containing lysine methyltransferase 7 (SETD7), alternatively referred to as KMT7, SET7/9, or KIAA1717, was first identified as a monomethyltransferase of K4 on histone H3 (8). The residue methylated by SETD7 commonly follows the consensus amino acid motif [K/R]-[S/T/A]-[K] (9). Accordingly, several essential proteins involved in tumor progression were also discovered to be non-histone substrates of SETD7, including the tumor suppressor p53 (10, 11), nuclear factor kB (NF-kB) p65 (12), hypoxia-induced factor-1a (HIF-1a) (13), *β*-catenin (14), and the proto-oncogene KRAS (15). The methylation of proteins mediated by SETD7 has a wide range of outcomes. While methylation of p53 (K372) stabilized this protein and increased transcriptional activity (10), methylation of *β*-catenin (K180) or KRAS (K182 and K184) by SETD7 promoted ubiquitination and proteasomal degradation (14, 15). Because SETD7 can positively or negatively regulate multiple proteins, its role in tumor progression may vary depending on the substrate and the cellular context (16). Recently, SETD7-mediated methylation of ATG16L1, an autophagy-related (ATG) protein, has been shown to act as a new regulatory mechanism to govern the formation of autophagosome (17). However, no other ATG proteins have been identified as substrates of SETD7 so far.

Here, we report that SETD7 interacts with the autophagy protein LC3B. SETD7 methylates LC3B at K51 and promotes LC3B ubiquitination and degradation, resulting in reduced formation of autophagosomes. Moreover, SETD7 plays a tumor-promotive function in ovarian cancer (OC) cells in a K51 methylation-dependent manner. Collectively, our findings uncover a novel modification of LC3B and highlight the oncogenic role of SETD7 *via* mediating LC3B methylation and degradation.

## Results

### SETD7 is a potential oncogene in ovarian cancer cells

**Figure 1:**
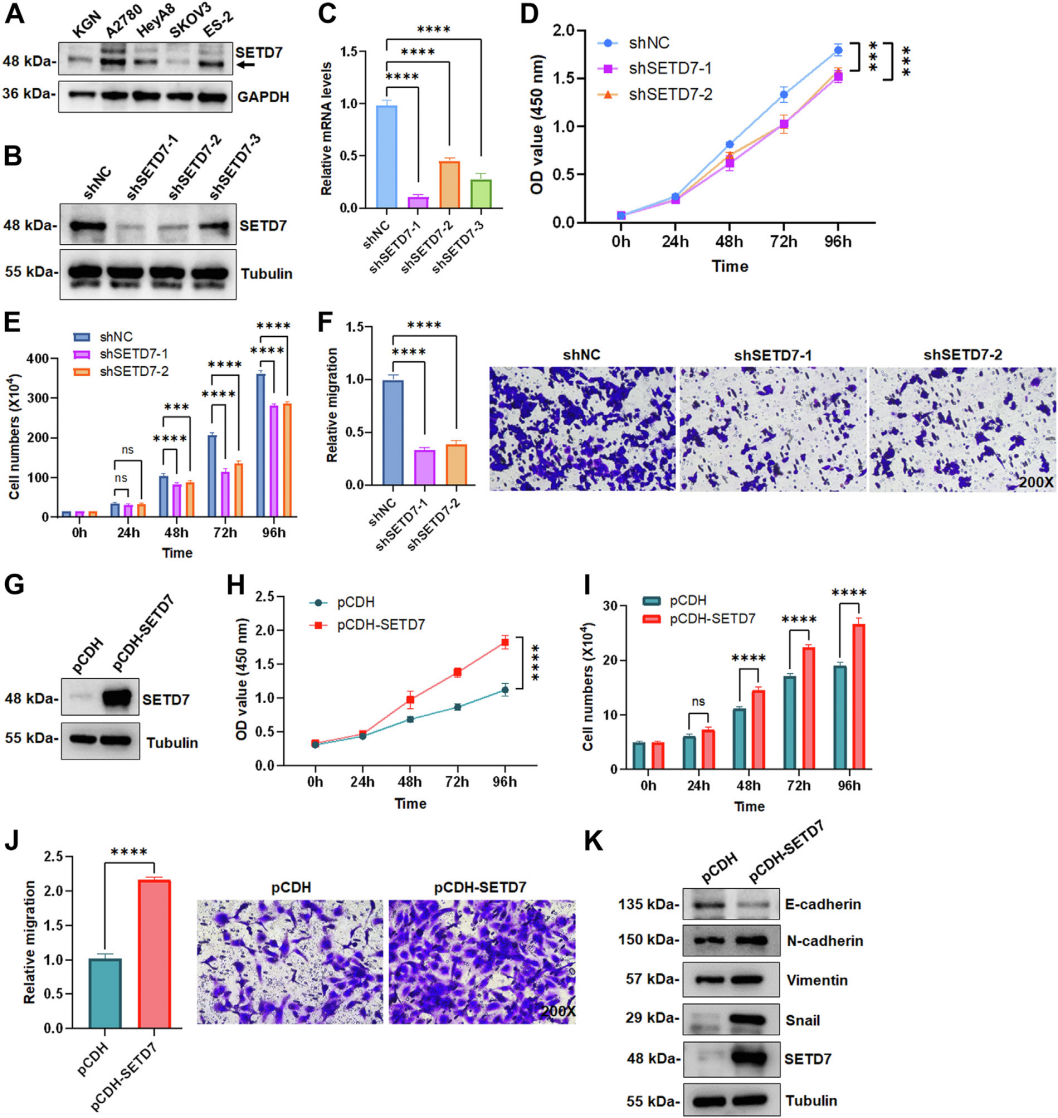
**SETD7 plays a tumor-promotive role in OC cells.***A*, the expression levels of SETD7 protein in four OC cell lines and one ovarian granulosa cell line (KGN) were analyzed by Western blot. *B*, Western blot was performed for SETD7 in A2780 cells infected with shSETD7-1, shSETD7-2, shSETD7-3, or negative control shRNA (shNC). *C*, the mRNA levels of *SETD7* in A2780 cells infected with shSETD7-1, shSETD7-2, shSETD7-3, or shNC were determined using real-time PCR. Each bar represents the mean ± SD (*n* = 3). ∗∗∗∗*p* < 0.0001 (One-way ANOVA followed by Dunnett’s multiple comparison). *D*, knockdown of SETD7 (SETD7 KD) inhibited the proliferation of A2780 cells determined by the CCK-8 assay. Each bar represents the mean ± SD (*n* = 3). ∗∗∗*p* < 0.001 (Two-way ANOVA followed by Tukey’s multiple comparison). *E*, SETD7 KD inhibited the proliferation of A2780 cells determined by cell counting assay. Each bar represents the mean ± SD (*n* = 3). ns: not significant (*p* > 0.05), ∗∗∗*p* < 0.001, ∗∗∗∗*p* < 0.0001 (Two-way ANOVA followed by Tukey’s multiple comparison). *F*, SETD7 KD inhibited the migration of A2780 cells determined by the Transwell assay. *Left panels* provide a graphical representation of the accumulated number of migrated cells at 48 h, and the *right panels* show the representative images (200 × magnification). Each bar represents the mean ± SD (*n* = 3). ∗∗∗∗*p* < 0.0001 (One-way ANOVA followed by Dunnett’s multiple comparison). *G*, Western blot for SETD7 in SKOV3 cells infected with pCDH-SETD7 or control vector (pCDH). *H*, overexpression of SETD7 (SETD7 OE) promoted the proliferation of SKOV3 cells determined by the CCK-8 assay. Each bar represents the mean ± SD (*n* = 3). ∗∗∗∗*p* < 0.0001 (Two-way ANOVA followed by Dunnett’s multiple comparison). *I*, SETD7 OE promoted the proliferation of SKOV3 cells determined by cell counting assay. Each bar represents the mean ± SD (*n* = 3). ns: not significant (*p* > 0.05), ∗∗∗∗*p* < 0.0001 (Two-way ANOVA followed by Dunnett’s multiple comparison). *J*, SETD7 OE promoted the migration of SKOV3 cells determined by the Transwell assay. *Left panels* provide a graphical representation of the accumulated number of migrated cells at 24 h, and the *right panels* show the representative images (200 × magnification). Each bar represents the mean ± SD (*n* = 3). ∗∗∗∗*p* < 0.0001 (Student’s *t* test). *K*, SETD7 OE SKOV3 cells were subjected to Western blot to test the expression of SETD7, E-cadherin, N-cadherin, Vimentin, and Snail, taking α-tubulin as an internal reference.

To understand the role of SETD7 in OC progression, we first examined the expression of SETD7 protein in OC cell lines. In comparison to the KGN cell line, the expression levels of SETD7 were higher in the A2780, HeyA8, and ES-2 cell lines and lower in the SKOV3 cell line (Fig. 1*A*). We next performed lentivirus-mediated knockdown of SETD7 (SETD7 KD) in the A2780 cell line using SETD7-specific shRNAs or overexpression of SETD7 cDNA (SETD7 OE) in the SKOV3 cell line. While SETD7 KD significantly impaired cell proliferation and migration (Figs. 1, *B–F* and S1*A*), SETD7 OE promoted these processes (Figs. 1, *G–J* and S1*B*). To further investigate the pro-tumor effect of SETD7, we assessed the expression of epithelial-mesenchymal transition (EMT) markers. As expected, Snail and N-cadherin were noticeably elevated, while E-cadherin was decreased in SETD7 OE SKOV3 cells (Fig. 1*K*). Overall, these results imply a tumor-promotive effect of SETD7 in OC.

### SETD7 promotes LC3B ubiquitination and degradation

**Figure 2:**
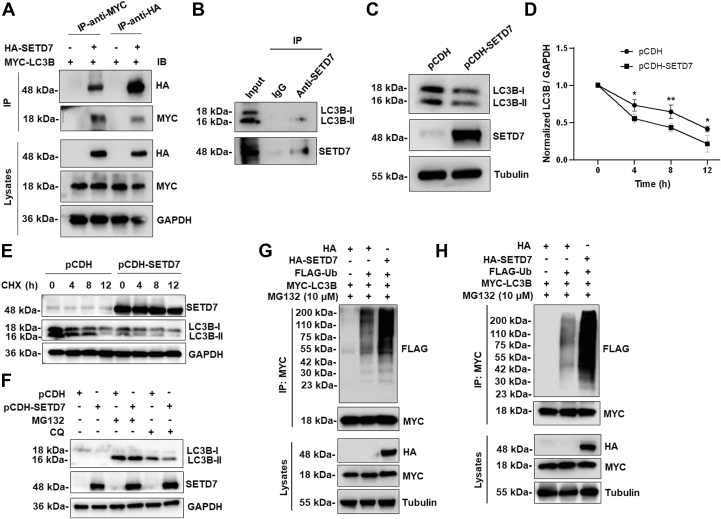
**LC3B is proteasomally degraded *via* SETD7.***A*, LC3B or SETD7 was co-immunoprecipitated with SETD7 or LC3B, respectively, in HEK293T cells co-expressing MYC-tagged LC3B and HA-tagged SETD7. *B*, endogenous SETD7 interacted with endogenous LC3B in HEK293T cells. *C*, stably expressed control vector (pCDH) or pCDH-SETD7 SKOV3 cells were generated. The lysates were immunoblotted with the antibodies indicated on the *right*. The Tubulin panel is reused from Figure 1*K* as both panels originate from the same experimental blot. *D* and *E*, stability of endogenous LC3B protein was determined by Western blot in HEK293T cells stably expressing pCDH or pCDH-SETD7, followed by 36 μM cycloheximide (CHX) treatment for the indicated times. Summarized data of LC3B half-life are shown (*D*), and representative immunoblot images of LC3B levels are indicated (*E*). Each bar represents the mean ± SD (*n* = 3). ∗*p* < 0.05, ∗∗*p* < 0.01 (Two-way ANOVA followed by Tukey’s multiple comparison). *F*, LC3B protein levels were determined by Western blot in HEK293T cells stably expressing pCDH or pCDH-SETD7 in the presence of 10 μM MG132 or 50 μM chloroquine (CQ) for 10 h. *G* and *H*, HEK293T (*G*) or SKOV3 cells (*H*) were co-transfected with FLAG-tagged ubiquitin (Ub), HA-tagged SETD7, or empty vector pCMV3-N-HA (HA), together with MYC-tagged LC3B in the presence of 10 μM MG132 for 10 h. The ubiquitination of LC3B was determined by anti-MYC immunoprecipitation followed by immunoblotting with anti-FLAG antibody.

To clarify the molecular mechanism of SETD7 in OC oncogenesis, we first ascertained the connection between SETD7 and LC3B. We introduced HA-SETD7 or HA empty vector into LC3B overexpressing HEK293T cells. Immunoprecipitation analysis showed that HA-SETD7 or MYC-LC3B could pull down MYC-LC3B or HA-SETD7 (Fig. 2*A*), respectively. To validate if these proteins interact endogenously, we employed a polyclonal antibody against SETD7 to conduct the immunoprecipitation assay in HEK293T cells. Our results demonstrated that endogenous SETD7 interacted with endogenous LC3B (Fig. 2*B*). To further determine the mechanism of the interaction between SETD7 and LC3B, we tested if SETD7 impinges on LC3B transcription. The results from real-time PCR indicated that *LC3B* mRNA expression was not altered by either SETD7 OE in SKOV3 cells (Fig. S2*A*) or SETD7 KD in A2780 cells (Fig. S2*B*). Interestingly, SETD7 OE obviously reduced LC3B protein levels (Fig. 2*C*), suggesting that SETD7 may influence LC3B protein stability. To examine the impact of SETD7 OE on this process, we performed a CHX chase analysis of the degradation of LC3B in wild-type (WT) and SETD7 OE cells. We found that SETD7 OE accelerated the degradation of endogenous LC3B protein (Fig. 2, *D* and *E*). Both the autophagy process and the ubiquitin-proteasome system (UPS) can break down proteins in mammalian cells (18). By employing inhibitors of these distinct protein degradation pathways, we found that proteasome inhibitor MG132 could completely rescue the degradation of LC3B mediated by SETD7, whereas lysosome inhibitor CQ only partially reversed this degradation (Fig. 2*F*), implying that LC3B is primarily degraded through the UPS. To further investigate ubiquitination in LC3B regulation, we transfected HA-SETD7 or FLAG-ubiquitin (Ub) into HEK293T and SKOV3 cells overexpressing MYC-LC3B. LC3B was heavily ubiquitinated when co-expressed with SETD7 (Fig. 2, *G* and *H*). These data show that SETD7 promotes the ubiquitination of LC3B, targeting it for degradation by the UPS.

### SETD7 methylates LC3B at K51

**Figure 3:**
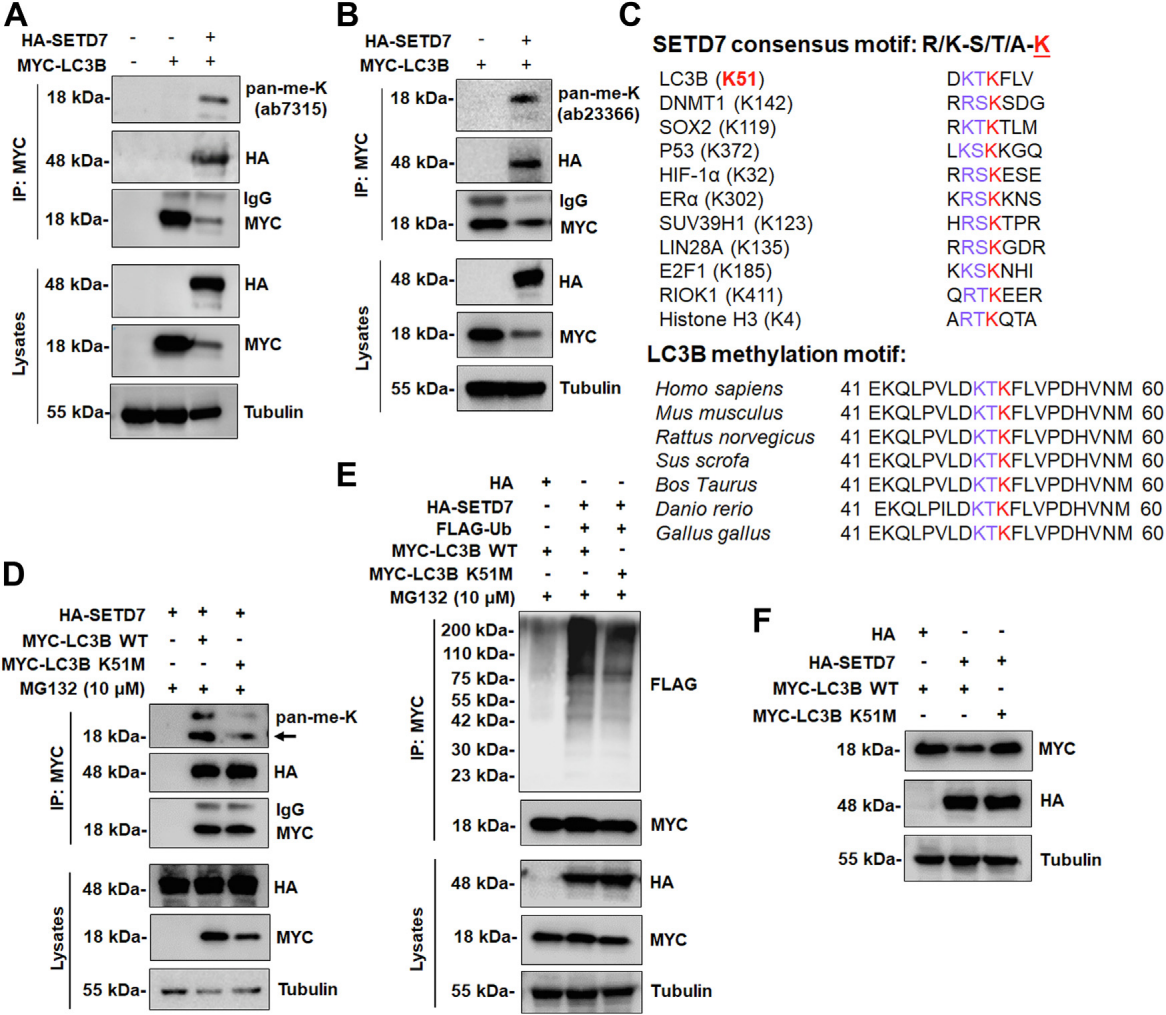
**SETD7 methylates LC3B at K51, leading to LC3B ubiquitination and degradation.***A* and *B*, HEK293T cells were co-transfected with HA-tagged SETD7 or MYC-tagged LC3B. LC3B was immunoprecipitated using anti-MYC antibody and the methylation levels of LC3B were detected by using dimethyl-lysine (ab7315) (*A*), or methyl-lysine (mono-, dimethylated) (ab23366) antibody (*B*). *C*, alignment of the consensus amino acid residues adjacent to lysine targeted by SETD7, and the identification of a putative SETD7 methylation site in LC3B. The consensus SETD7 recognition sequence, the lysines targeted for methylation by SETD7 in known substrates, and the methylated lysine are shown in *red* in each case. *D*, transiently expressed HA-tagged SETD7 HEK293T cells were transfected with MYC-tagged LC3B wild-type (WT) or mutant with methionine (M) as a mimic of the nonmethylated lysine (K51M). LC3B methylation was detected by using a dimethyl-lysine (ab7315) antibody and immunoprecipitated by using an anti-MYC antibody. Arrow indicates the target protein band. *E*, HEK293T cells were co-transfected with HA-tagged ETD7, empty vector pCMV3-N-HA (HA), FLAG-tagged Ub, and MYC-tagged LC3B WT or K51M mutant in the presence of 10 μM MG132 for 10 h. The ubiquitination of LC3B was determined by anti-MYC immunoprecipitation followed by Western blot with anti-FLAG antibody. *F*, HEK293T cells were transfected with the indicated combinations of HA-tagged SETD7, empty vector pCMV3-N-HA (HA), and MYC-tagged LC3B WT or K51M mutant. The lysates were immunoblotted with the antibodies indicated on the *right*.

Since SETD7 has been demonstrated to methylate several nonhistone proteins (15, 17), we postulated that LC3B was methylated by SETD7, which subsequently caused LC3B to be ubiquitinated and degraded. To test this idea, we investigated if SETD7 interacted with methylated LC3B in SETD7 OE HEK293T cells. When LC3B was immunoprecipitated and probed with two different anti-methyl lysine antibodies (ab7315 antibody preferentially recognizing dimethylated lysine, and ab23366 antibody recognizing both mono- and dimethylated lysine), we found that LC3B was mono- or dimethylated in SETD7 OE cells but not in empty vector cells (Fig. 3, *A* and *B*). We then sought to determine the SETD7-methylated residue(s) in LC3B. The amino acid sequences of LC3B were compared with the sequences surrounding the SETD7 methylation sites of several known substrates (Fig. 3*C*). The conserved lysine residue at position 51 and its adjacent residues in LC3B were found to conform to the consensus SETD7 recognition sequence, which is denoted by a K/R-S/T/A-**K** motif (where the methylated lysine site is highlighted) (9). When the full amino acid sequences of LC3B protein from different species were aligned, the LC3B methylation sequences were evolutionarily conserved (Fig. 3*C*). Furthermore, the LC3B mutant with methionine (M) as a mimic of the nonmethylated lysine (K51M) partially abolished LC3B methylation by SETD7 in HEK293T cells (Fig. 3*D*). Intriguingly, the LC3B K51M mutant showed reduced ubiquitination levels (Fig. 3*E*) and was resistant to SETD7-mediated degradation (Fig. 3*F*) as compared to LC3B WT, suggesting that methylation at K51 is critical for LC3B degradation (Fig. 2*G*). Together, these findings suggest that methylation of LC3B at the K51 residue by SETD7 promotes ubiquitination and degradation.

### Methylation of LC3B inhibits autophagy and facilitates cell proliferation and migration

**Figure 4:**
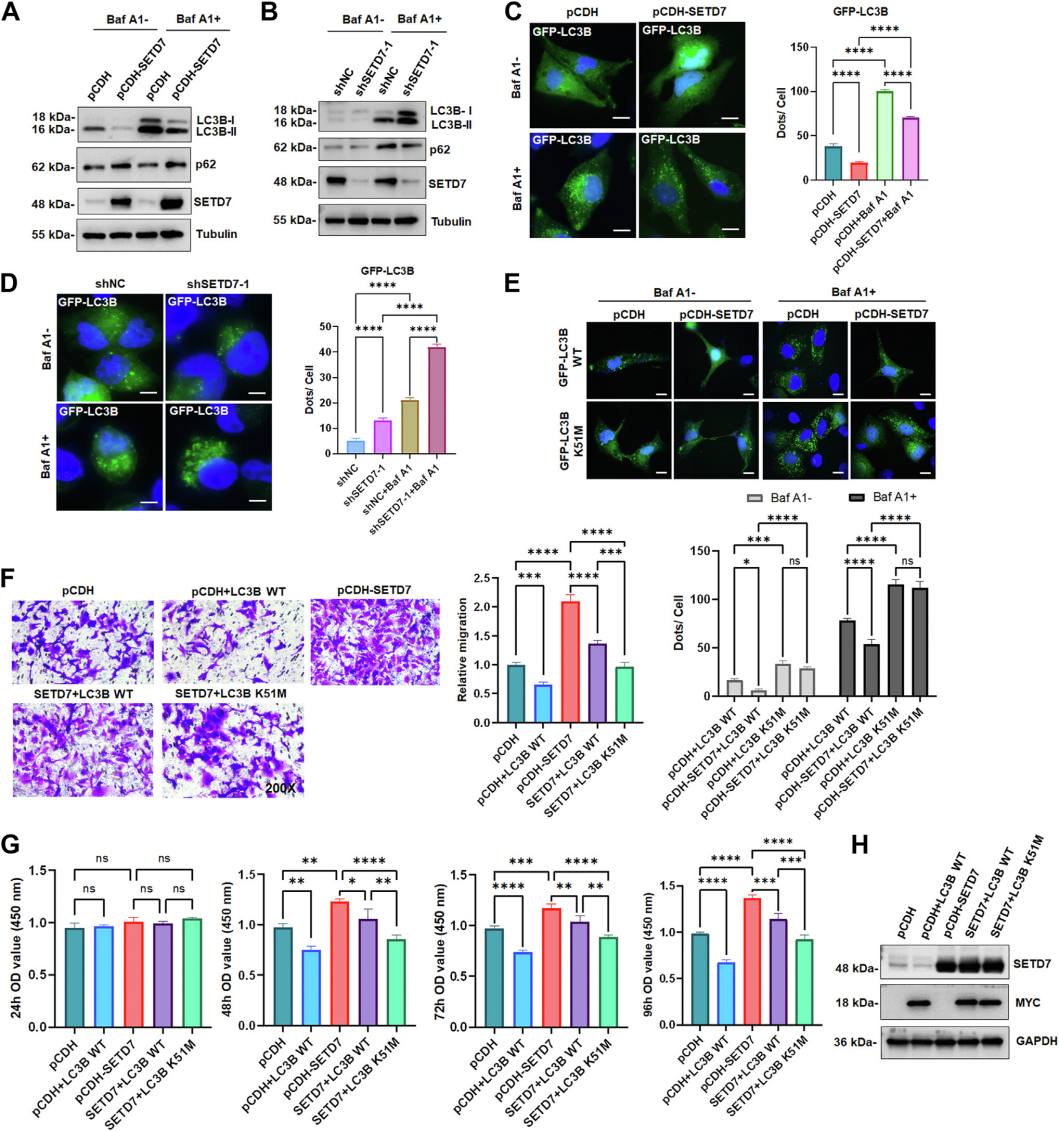
**Methylation of LC3B inhibits autophagy and promotes cell proliferation and migration.***A*, SKOV3 cells were stably transfected with control vector (pCDH) or pCDH-SETD7 in the absence or presence of bafilomycin A1 (Baf A1, 100 nM) for 10 h. The lysates were immunoblotted with the antibodies indicated on the *right*. *B*, A2780 cells were transfected stably with negative control (shNC) or shSETD7-1 in the absence or presence of Baf A1 (100 nM) for 10 h. The lysates were immunoblotted with the indicated antibodies. *C*, SKOV3 cells stably expressing pCDH or pCDH-SETD7 were transfected with GFP-LC3B in the absence or presence of Baf A1 (100 nM) for 10 h. *Left panels* show the representative images of LC3B puncta, and the *right panels* show the quantification of LC3B puncta. Scale bars, 10 μm. Error bars represent the mean ± SD (*n* = 3). ∗∗∗∗*p* < 0.0001 (One-way ANOVA followed by Tukey’s multiple comparison). *D*, A2780 cells that stably express shNC or shSETD7-1 were transfected with GFP-LC3B in the absence or presence of Baf A1 (100 nM) for 10 h. *Left panels* show the representative images of LC3B puncta, and the *right panels* show the quantification of LC3B puncta. Scale bars, 10 μm. Error bars represent the mean ± SD (*n* = 3). ∗∗∗∗*p* < 0.0001 (One-way ANOVA followed by Tukey’s multiple comparison). *E*, SKOV3 cells stably expressing pCDH or SETD7 were transfected with GFP-LC3B WT or K51M mutant in the absence or presence of Baf A1 (100 nM) for 10 h. *Top panels* show the representative images of LC3B puncta, and the *bottom panels* show the quantification of LC3B puncta. Scale bars, 10 μm. Error bars represent the mean ± SD (*n* = 3). ns: not significant (*p* > 0.05), ∗*p* < 0.05, ∗∗∗*p* < 0.001, ∗∗∗∗*p* < 0.0001 (Two-way ANOVA followed by Tukey’s multiple comparison). *F*, SKOV3 cells stably expressing pCDH or pCDH-SETD7 were transfected with MYC-LC3B WT or K51M mutant followed by a Transwell assay for 24 h. *Left panels* show the representative images of migrated cells, and the *right panels* show the quantification of migrated cells (200 × magnification). Error bars represent the mean ± SD (*n* = 3). ∗∗∗*p* < 0.001, ∗∗∗∗*p* < 0.0001 (One-way ANOVA followed by Tukey’s multiple comparison). *G*, SKOV3 cells stably expressing pCDH or pCDH-SETD7 were transfected with MYC-LC3B WT or K51M mutant followed by the CCK8 assay every 24 h. Error bars represent the mean ± SD (*n* = 3). ns: not significant (*p* > 0.05), ∗*p* < 0.05, ∗∗*p* < 0.01, ∗∗∗*p* < 0.001, ∗∗∗∗*p* < 0.0001 (One-way ANOVA followed by Tukey’s multiple comparison). *H*, Western blot was performed with the indicated antibodies in SKOV3 cells treated as in *F* and *G*.

We next investigated the function of LC3B methylation in OC cells. Since LC3B is required for initiating autophagosome biogenesis (19), we postulated that methylation of LC3B by SETD7 might regulate autophagy. As expected, attenuation of LC3B-II and accumulation of p62, which can be explained by decreased autophagic flux, were detected in SETD7 OE SKOV3 cells (Fig. 4*A*). Following administration of the autophagy inhibitor bafilomycin A1 (Baf A1), the accumulation of LC3B-II was increased (Fig. 4*A*). Conversely, the accumulation of LC3B-II and attenuation of p62 in response to Baf A1 were detected in SETD7 KD A2780 cells (Fig. 4*B*), suggesting that SETD7 blocks the autophagic flux. To further validate the effect of SETD7 on autophagy inhibition, we introduced GFP-LC3B into SKOV3 cells stably expressing SETD7. The number of green puncta in the SETD7 OE cells was significantly less than that in the control cells, and this tendency was further enhanced upon treatment with the autophagy inhibitor Baf A1 (Fig. 4*C*). Conversely, more green puncta accumulated in both untreated and Baf A1-treated SETD7 KD cells (Fig. 4*D*). Next, we determined the role of LC3B methylation on K51 induced by SETD7 in the suppression of autophagy. As shown in Figure 4*E*, SETD7 OE significantly reduced Baf A1-induced autophagy. The K51M mutant, but not WT LC3B, could reverse the decreased autophagy, further validating that the influence of SETD7 on autophagy was primarily due to the methylation of LC3B at K51.

Given that SETD7 promoted OC cell proliferation and migration (Fig. 1), we further investigated whether SETD7-mediated cell phenotypes depended on the methylation of LC3B. As shown in Figure 4*F*, the promotive effect of cell migration by SETD7 OE in SKOV3 cells was reversed by the LC3B K51M mutant. Accordingly, SETD7 OE-induced promotion of cell proliferation was rescued by the K51M mutant (Fig. 4, *G* and *H*). Taken together, these data suggest that methylation of LC3B hinders autophagy and promotes cell proliferation and migration.

## Discussion

**Figure 5:**
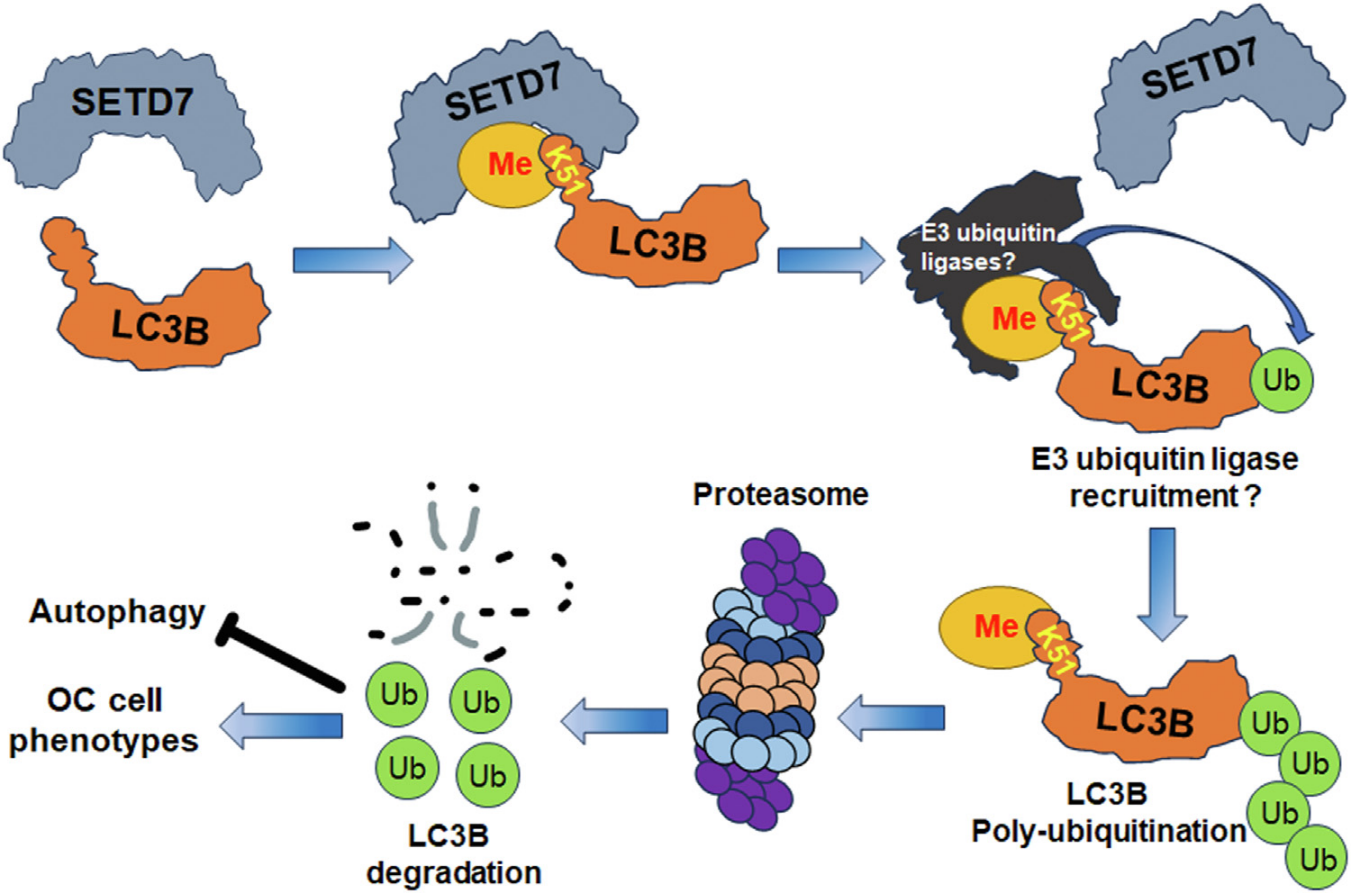
**Proposed model to describe the role of LC3B methylation by SETD7 in promoting LC3B degradation.** SETD7 methylates LC3B at lysine 51 (K51). This methylation destabilizes LC3B *via* ubiquitin-mediated protein degradation. Through the degradation of LC3B, SETD7 inhibits autophagy and promotes the proliferation and migration of OC cells.

Here, we revealed that the lysine residue in the LC3B protein is methylated by the lysine methyltransferase SETD7. SETD7 interacts with and dimethylates LC3B at K51. This methylation of LC3B increases its ubiquitination, which destabilizes the protein. Through the destabilization of the LC3B protein, SETD7 suppresses the autophagic flux and facilitates the proliferation and migration of OC cells (Fig. 5). These findings suggest that SETD7 methylates and destabilizes the autophagy protein LC3B, likely through the UPS, providing a mechanism by which SETD7 destabilizes LC3B and inhibits autophagy in OC cells.

PTMs of the human LC3 isoforms (LC3A, LC3B, and LC3C) are essential for the regulation of autophagy. Phosphorylation is the major modification of the LC3 isoforms throughout the autophagy process. Autophagy inhibition relies on the phosphorylation of LC3A at serine 12 (S12) by protein kinase A (20). The kinase STK4 phosphorylates LC3B at T50 to regulate the interaction of LC3B with FYCO1 and facilitate autophagosome–lysosome fusion (6, 21, 22). Strikingly, in addition to STK4-mediated regulation, NEK9 and protein kinase C may also phosphorylate LC3B T50 (22, 23). Accordingly, NEK9-mediated LC3B phosphorylation suppresses selective autophagy of p62 (22). Besides phosphorylation, there have been documented in other PTMs of LC3B, including ubiquitination and acetylation. Proautophagic K51-linked monoubiquitination of LC3B is mediated by UBA6 and BIRC6 (3). The acetyltransferases p300 and CBP mediate the acetylation of LC3B at K49 and K51 to prevent autophagosome formation, while SIRT1 deacetylates LC3B to remove it (4, 24).

Here, we identified the methylation of LC3B at K51 mediated by SETD7. This methylation facilitated the ubiquitination and degradation of LC3B and thereby inhibited autophagic flux. Our data provided direct evidence of the SETD7-mediated methylation and ubiquitination of LC3B *via* manipulating SETD7; however, it is unclear how methylation of LC3B affects its ubiquitination and which ubiquitin ligase mediates the ubiquitination of LC3B. Thus, further elucidation of the interaction between K51 methylation and ubiquitination is necessary to comprehend how different PTM types collaborate to modulate LC3B protein stability. Besides, the autophagy regulatory function of LC3B methylation in OC cells was also investigated. Our results provide new information on LC3B modification and its function in autophagy regulation.

SETD7 is a significant modifier of multiple non-histone proteins and exerts either tumor-suppressive or oncogenic functions in various cancer types (16). According to some research, SETD7 functions as a tumor suppressor in breast cancer (25), renal cell carcinoma (26), colorectal cancer, and gastric cancer (27). However, others have noted an oncogenic role for SETD7 in prostate cancer (28), hepatocellular carcinoma (29), and intestinal tumorigenesis (30). The controversial roles of SETD7 in various cancer types imply that the effects of SETD7 on cancer development depend on its interacting proteins and the particular cellular context. We found that SETD7 promoted OC cell proliferation and migration through methylating LC3B. The oncogenic effect of SETD7 was reversed by the methylation-deficient mutant LC3B K51M. Our data suggest that SETD7 plays a potent tumor-promotive role in OC cells *via* modulating LC3B methylation and degradation. Although LC3B has been reported to undergo PTMs such as acetylation, phosphorylation, and ubiquitination (3, 4, 21), it is still unclear whether the methylation of LC3B can affect these PTMs and its function. Additionally, the current study revealed that the methylation of LC3B promoted its ubiquitination and degradation, which in turn inhibited autophagic flux. Nevertheless, whether the SETD7-mediated LC3B methylation facilitates OC cell phenotypes through autophagy inhibition remains elusive. Therefore, further research is required to fully characterize the PTMs of LC3B in autophagy regulation, which may offer promising targets for OC treatment.

In summary, we have identified a methylation-dependent regulatory mechanism controlling LC3B’s role in autophagy for the first time, and the methylation of LC3B can inhibit autophagosome formation and promote cancer cell phenotypes in OC cells. Our data open a potential novel paradigm of reversible LC3B methylation in affecting autophagy.

## Experimental procedures

### Cell lines and cell culture

Human OC cell lines A2780, SKOV3, Hey A8, and ES-2, ovarian granulosa cell line KGN, and HEK293T were obtained from the American Type Culture Collection (ATCC) and STR-authenticated by Shanghai Biowing Applied Biotechnology Co. LTD. The A2780 cell line originated from undifferentiated ovarian endometrioid adenocarcinoma. The SKOV3, Hey A8, and ES-2 cell lines originated from poorly differentiated ovarian serous cystadenocarcinoma. Every cell line was cultivated in monolayers using the proper media: A2780 cells were grown in RPMI 1640 medium (350–030-CL; Wisent Biotechnology); SKOV3 and ES-2 cells were grown in McCoy's 5A medium (317–010-CL; Wisent Biotechnology); KGN, Hey A8, and HEK293T cells were grown in Dulbecco’s Modified Eagle Medium (319–015-CL; Wisent Biotechnology) supplemented with 10% fetal bovine serum (Gibco) and 100 U/ml penicillin-streptomycin (Life Technologies). All cells were kept in an atmosphere of 5% CO_2_ at 37 °C.

### Antibodies and reagents

SETD7 (24840-1-AP), FLAG (20543-1-AP), E-cadherin (20874-1-AP), and Snail (13099-1-AP) antibodies were purchased from Proteintech. P62 (sc-48402), N-cadherin (sc-59987), LC3B (sc-376404), and HA (sc-57592) antibodies were provided by Santa Cruz Biotechnology. Vimentin (5741) antibody was purchased from Cell Signaling Technology. Tubulin (BS1699) and GAPDH (AP0063) antibodies were obtained from Bioworld Technology. Pan methylation (ab7315) and histone H3 (ab1791) antibodies, and MG132 (ab141003) were obtained from Abcam. MYC (MMS-150R) antibodies were purchased from Covance. Mouse (31,430) and Rabbit (31,460) HRP-conjugated secondary antibodies were obtained from Thermo Fisher Scientific. Lipofectamine 2000 (11,668,019) and puromycin (A11138–03) were purchased from Invitrogen. Polybrene (H9268), cycloheximide (CHX, C7698), chloroquine (CQ, C6628), bafilomycin A1 (Baf A1, 88,899–55–2), aprotinin (A3886), and leupeptin (L2884) were from Sigma-Aldrich. 4,6-diamidino-2-phenylindole (DAPI, C1005) was purchased from Beyotime Biotechnology. Transwell chamber (TCS020024) was purchased from Jet Bio-Filtration. Cell Counting Kit (CCK)-8 (A311–01) was purchased from Vazyme Biotech.

### Plasmids

Flag-ubiquitin plasmid (HH-gene-433) was purchased from HedgehogBio Science and Technology Ltd (Shanghai, China). The plasmids pCDH-CMV-MCS-EF1-Puro lentiviral vector (LM-1480, LMAI Bio), pGEX-4T-3-LC3 and pcDNA3-MYC-LC3 (gifts from Dr Wannian Yang Lab), and pAcGFP1 (a gift from Dr Likai Ji at Jiangsu University) were used. Certain plasmids were repurposed into other target vectors. The full-length human *SETD7* cDNA (GenBank accession number NM_030648) sequences were amplified by DNA polymerase from a human mRNA pool generated by RT-PCR using the PrimeScript RT Reagent kit (Takara Bio). The *SETD7* cDNA was cloned into either the EcoRI/BamHI site of pCDH-CMV-MCS-EF1-Puro (LM-1480, LMAI Bio) or the HindIII/XbaI site of pCMV3-N-HA (CV017, Sino Biological) vectors. Site-directed mutagenesis of MYC-tagged *LC3B* or GFP-*LC3B* was performed using the DNA Mutagenesis Kit (200518, Stratagene). The *LC3B* K51M cDNA was cloned into either the BamHⅠ/EcoRI site of pcDNA3-MYC or the NheI/BamHI site of pAcGFP1. All the sequences were confirmed by Sanger sequencing (Synbio Technologies, Suzhou, China; Supplementary Information). To create stable *SETD7* knockdown in A2780 cells, the AgeI/EcoRI site of the pLKO.1 vector (8453, Addgene) was targeted with *SETD7* shRNAs (shSETD7-1#, GGGAGTTTACACTTACGAAGA; shSETD7-2#, GGACCGCACTTTATGGGAAAT; shSETD7-3#, GCAAACTGGCTACCCTTATGT). A scrambled shRNA sequence (shNC: CCTAAGGTTAAGTCGCCCTCG; 136,035, Addgene) with no homology to any known human gene was used as a negative control.

### Real-time PCR

Total RNA was extracted using the RNAiso plus and reverse transcribed into cDNA using the PrimeScript RT Reagent kit (Takara Bio) according to the manufacturer’s instructions. The cDNA was then subjected to real-time PCR as previously mentioned (31). All real-time PCR reactions were performed on a Bio-Rad CFX96 system (Bio-Rad) using the SYBR-Green PCR Master Mix (Takara Bio) according to the product specification. The primer sequences were as follows: *SETD7* (GenBank accession no. NM_001306200), 5′-TCACCTACTCCTCCACAGAC-3’ (forward) and 5′-TCATCCACATAATACCCCTCCAG-3’ (reverse); *LC3B* (GenBank accession no. NM_022818.5), 5′-CCATGTCCCTGCACCATG-3’ (forward) and 5′-CTGCTTCTCACCCTTGTATCG-3’ (reverse); and *GAPDH* (GenBank accession no. NM_001256799.3), 5′-CACCAGGGCTGCTTTTAACTC-3’ (forward) and 5′-CTTGACGGTGCCATGGAATTTG-3’ (reverse). The PCR parameters were as follows: first denaturation at 95 °C for 30 s, then denaturation for 40 s at 95 °C for 5 s, annealing at 58 °C for 30 s, and elongation at 72 °C for 60 s. With *GAPDH* serving as the reference gene, the relative results were examined using the comparative cycle threshold approach (2^−ΔΔCt^).

### Western blot and immunoprecipitation

Cells were rinsed with ice-cold phosphate-buffered saline (PBS) and lysed with the radioimmunoprecipitation assay (RIPA, sc-24948, Santa Cruz Biotechnology) buffer supplemented with protease inhibitors, including aprotinin and leupeptin (Sigma-Aldrich). The cell lysates were centrifuged at 12,000 rpm for 15 min at 4 °C. The protein concentrations were measured by colorimetric detection using a bicinchoninic acid (BCA) assay (Thermo Fisher Scientific, MA, USA). The proteins (30 μg) were equally resolved using SDS-polyacrylamide gel electrophoresis (SDS-PAGE) and then immunoblotted with the specified antibodies. For immunoprecipitation, the lysates (1000–3000 μg) were incubated with normal rabbit IgG (5 μg, A7016), mouse IgG (5 μg, A7028, Beyotime Biotechnology), anti-MYC (5 μg), or anti-HA (5 μg) antibodies at 4 °C for 60 min. Thereafter, the protein A/G beads (30 μl, P2019; Beyotime Biotechnology) were added to the supernatants and shaken for 8 h at 4 °C. Then, the immunoprecipitate was washed with lysis buffer and subsequently eluted with the SDS sample buffer. The proteins were separated by 10% SDS-PAGE and transferred to polyvinylidene fluoride (PVDF, Millipore) membranes. The membranes were then incubated with the primary antibodies: SETD7 (1:2000), LC3B (1:1000), MYC (1:1000), P62 (1:1000), E-cadherin (1:1000), Snail (1:1000), N-cadherin (1:1000), Vimentin (1:1000), Tubulin (1:10,000), GAPDH (1:1000), or Histone H3 (1:1000). Following a wash step, the membranes were incubated with peroxidase-bound anti-mouse or anti-rabbit IgG (1:2000) at 37 °C for 2 h and detected using Pierce ECL Western blotting substrate (Thermo Fisher Scientific).

### Ubiquitination assay

Ubiquitylation assay was performed as previously described (32). HEK293T or SKOV3 cells were co-transfected with FLAG-tagged Ub, HA-tagged SETD7, or the empty vector pCMV3-N-HA, together with MYC-tagged LC3B, in the presence of 10 μM MG132 for 10 h. The ubiquitylation status of exogenous LC3B was assessed by anti-MYC immunoprecipitation, followed by immunoblotting with an anti-FLAG antibody in RIPA lysis buffer (sc-24948; Santa Cruz Biotechnology).

### Immunofluorescence

The cells were grown in culture dishes with coverslips (BS-18-RC, Biosharp) to 60 to 80% confluence, and then fixed for 30 min at room temperature (RT) with 4% paraformaldehyde. Following overnight incubation at 4 °C with the primary antibody anti-LC3B (1:100) in PBS containing 5% bovine serum albumin (BSA), the samples were rinsed with PBS and then incubated with a secondary antibody (FITC/Alexa Fluor 488/594) in PBS containing 5% BSA for 1 h at RT in the dark. After three PBS washes, the samples were treated with DAPI for 5 min and then visualized under confocal microscopy (Zeiss LSM710, Jena).

### Establishment of stable SETD7 overexpressing cell line

To generate a cell line that stably overexpresses either pCDH or pCDH-SETD7, HEK293T cells cultured in 100 mm culture dishes were cotransfected with 0.5 μg of pHR'-CMV-VSVG, 1.5 μg of pHR'-CMV-8.2VPR, and 2 μg of pCDH-SETD7 or pCDH, using the Lipofectamine 2000 reagent. Subsequently, the supernatant containing the lentivirus was harvested at 48 and 72 h post-transfection. This supernatant was then clarified through filtration using a 0.45-μm membrane filter (Millipore) and concentrated *via* ultracentrifugation at 70,000*g* for 2 h at 4 °C (Avanti J-30I, Beckman Coulter, Brea, CA, USA). For infection, 1 ml of the lentiviral supernatant was added to SKOV3 cells in a 60 mm culture dish, along with 4 ml of growth medium supplemented with 6 μg/ml polybrene (Sigma-Aldrich). Following two rounds of infection, the SKOV3 cells underwent selection with 2 μg/ml puromycin for 3 days and then maintained for 1 week using 1 μg/ml puromycin. The stability of the clones was verified through Western blot analysis (33).

### Generation of stable SETD7 knockdown cell line

As described earlier (34), a stable cell line expressing SETD7 shRNAs or scrambled shRNA (shNC) was generated. Briefly, HEK293T cells that expressed high levels of SETD7 were cotransfected with 0.5 μg of pHR'-CMV-VSVG, 1.5 μg of pHR'-CMV-8.2VPR, and 2 μg of SETD7 shRNAs or shNC, utilizing the Lipofectamine 2000 reagent. The protocol for virus production and cell transfection adhered to the methodology outlined in the preceding section. The transduced cells were selected with 2 μg/ml puromycin for 1 week until stable clones were established. The levels of SETD7 knockdown were verified by real-time PCR and Western blot.

### Autophagy analysis

The rate of LC3B-II turnover and the quantity of exogenous LC3B puncta were used to assess autophagic flux and validated with the levels of p62 protein for autophagy completion. To estimate the LC3B-II turnover, cells were exposed to 100 nM bafilomycin A1 for 10 h, followed by harvesting the cells for Western blot analysis of LC3B-I and LC3B-II protein levels. To achieve exogenous LC3B labeling, cells were transfected with GFP-LC3B either with or without 100 nM bafilomycin A1 for 10 h and then stained with DAPI. The total number of cells in the images was determined by counting the stained nuclei. Autophagy was quantified by counting GFP-LC3B puncta under a confocal microscope (Zeiss LSM710, Jena, Germany).

### Cell proliferation assay

A2780 and SKOV3 cells (3–5 × 10^4^) were inoculated in 12-well plates and cultivated for the allotted time. Using a hemocytometer and a microscope with 100 × magnification, the cells in six randomly selected fields were counted after 0, 24, 48, 72, and 96 h. By comparing the increase in cell counts since seeding, cell proliferative activity was evaluated. Each specimen was analyzed twice, and three independent experiments were carried out.

### Cell growth analysis

Cell viability was assessed using the CCK-8 assay (Vazyme Biotech). A2780 and SKOV3 cells (3–5 × 10^4^) were introduced to the 96-well plates, and the cells were pre-cultured in the incubator for 24 h. Then, 100 μl of CCK-8 (10%) was added to each well, and cultured at 37 °C for 2 h. The absorbance was measured at 450 nm by a microplate reader (Bio-Rad 650).

### Transwell assay

Cell migration was evaluated as previously described (34). Serum-free medium containing A2780 and SKOV3 cells (6 × 10^3^) was placed into the upper Transwell chamber (Jet Bio-Filtration), while chemoattractant medium containing 20% FBS was added to the lower chamber. Following a 48-h incubation period at 37 °C and 5% CO_2_, the cells in the upper chamber were cleared using a cotton swab, and the cells on the lower layer were stained with 1% crystal violet for 15 min. Microscopical (200 × ) measurements of the migrated cells were carried out in five random fields, and the results were averaged.

### Wound-healing assay

A2780 and SKOV3 cells were seeded in the 6-well plates and cultivated until they reached 100% confluence. Mitomycin C (10 μg/ml) was administered for 2 h to prevent cell proliferation. The single layer of cells was scratched to create the artificial linear wounds using a 200-μL pipette tip. The width of the scratch gap was observed and photographed at 24 or 72 h using an inverted microscope (34). ImageJ software was used to measure the distance the cells traveled into the wound areas. The migration rate was calculated as follows: % of scratch closure = [(initial wound length) − (wound length at 24/72 h)]/(initial wound length) × 100.

### Statistical analysis

Statistical analysis was conducted with the Prism 8 (GraphPad Software). The significance between two or more groups was examined using the Student’s *t* test or ANOVA with the relevant statistical test, as shown in the figure legends. The results were expressed as mean ± standard deviation (SD), with *p* < 0.05 showing statistical significance.

## Data availability

All data generated or analyzed during this study are included in this published article and its supplementary information files.

## Supporting information

This article contains supporting information.

## Conflict of interests

The authors declare that they have no conflicts of interest with the contents of this article.

## Supporting information

Supplementary Figures

Plasmid Sequencing Data

Original Membranes for WB

Cell Line Authentication
