# Supplementary material for: SETD7 promotes LC3B methylation and degradation in ovarian cancer
Source: J Biol Chem. 2024 Dec 25;301(2):108134. doi: 10.1016/j.jbc.2024.108134 (PMC11791264; doi:10.1016/j.jbc.2024.108134)
Supplement: Supplementary Figures [file mmc1.docx]

**Supplementary Figures**

**SETD7 promotes LC3B methylation and degradation in ovarian cancer**

Ziwei Zhang, Mingyang Li, Yanan Hou, Ting Huang, Bowen Zhang, Qiong Lin, Genbao Shao*

Department of Basic Medicine, School of Medicine, Jiangsu University, Zhenjiang 212013, Jiangsu, China.

*Corresponding Author:

Dr. Genbao Shao, Department of Basic Medicine, School of Medicine, Jiangsu University, 301 Xuefu Road, Zhenjiang 212013, Jiangsu, China.

E-mail: gbshao07@ujs.edu.cn

**Short Title:** SETD7 methylates LC3B for degradation


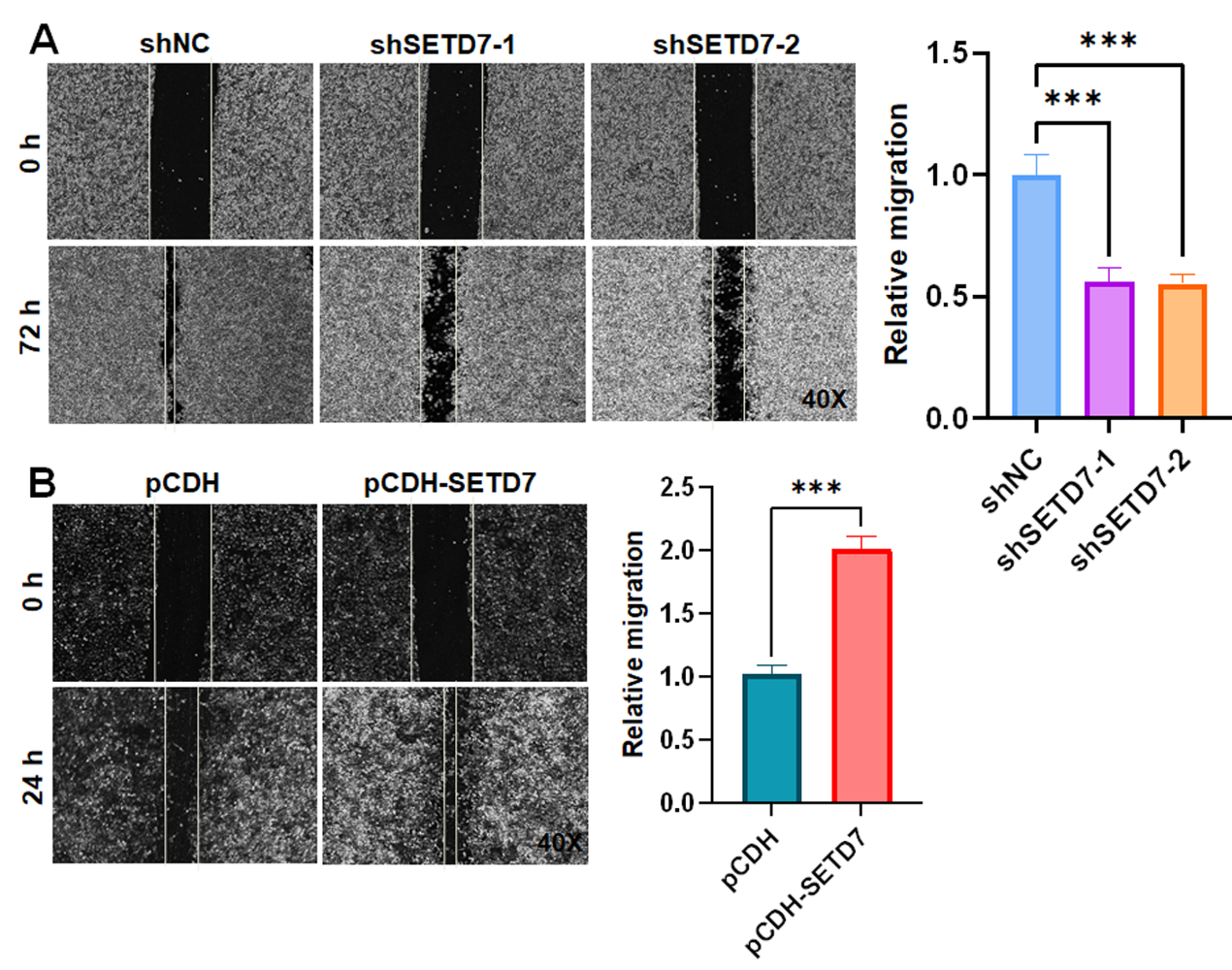


**Figure S1**. **SETD7 promotes cell migration.** *A*, the effects of SETD7 KD or negative control shRNA (shNC) on the migration of A2780 cells were determined by wound-healing assay. Left panels show the representative images (40× magnification), and the right panels provide a graphical representation of the accumulated number of migrated cells at 72 h. Each bar represents the mean ± SD (*n* = 3). ****p* < 0.001 (One-way ANOVA followed by Dunnett’s multiple comparison). *B*, the effects of SETD7 OE or control vector (pCDH) on the migration of SKOV3 cells were measured by a wound-healing assay. Left panels show the representative images (40× magnification), and the right panels provide a graphical representation of the accumulated number of migrated cells at 24 h. Each bar represents the mean ± SD (*n* = 3). ****p* < 0.001 (Student’s *t*-test).


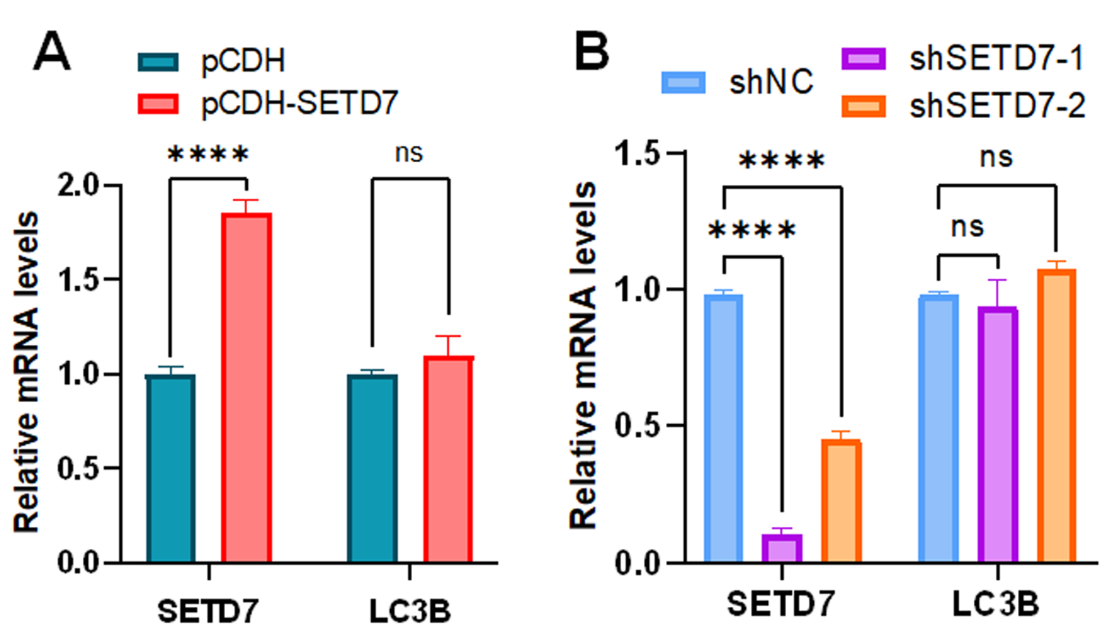


**Figure S2**. **SETD7 does not modify the expression of *LC3B* mRNA.** *A*, the mRNA levels of *LC3B* and *SETD7* were analyzed in SKOV3 cells that expressed either the control vector (pCDH) or pCDH-SETD7, using real-time PCR. Each bar represents the mean ± SD (*n* = 3). ns: not significant, *****p* < 0.0001 (Student’s *t*-test). *B*, the mRNA levels of *LC3B* and *SETD7* were analyzed in A2780 cells that expressed either the negative control (shNC), shSETD7-1, or shSETD7-2, using real-time PCR. Each bar represents the mean ± SD (*n* = 3). ns: not significant, *****p* < 0.0001 (One-way ANOVA followed by Dunnett’s multiple comparison).
