# Supplementary material for: SETD7 promotes LC3B methylation and degradation in ovarian cancer
Source: J Biol Chem. 2024 Dec 25;301(2):108134. doi: 10.1016/j.jbc.2024.108134 (PMC11791264; doi:10.1016/j.jbc.2024.108134)

Supplementary Information

1. pCDH-CMV-MCS-EF1-Puro-SETD7

(1) Plasmid map:

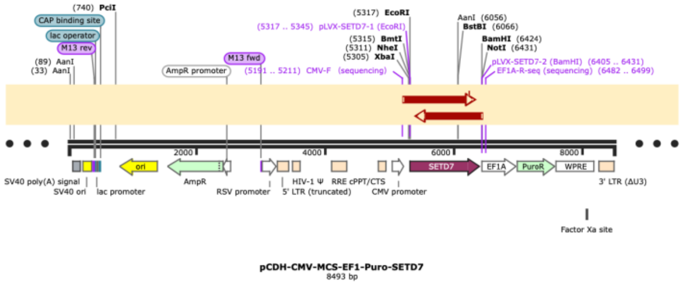

(2) Sequencing data:

Prime 1: CMV-F

Prime 2: EF1A-R-seq

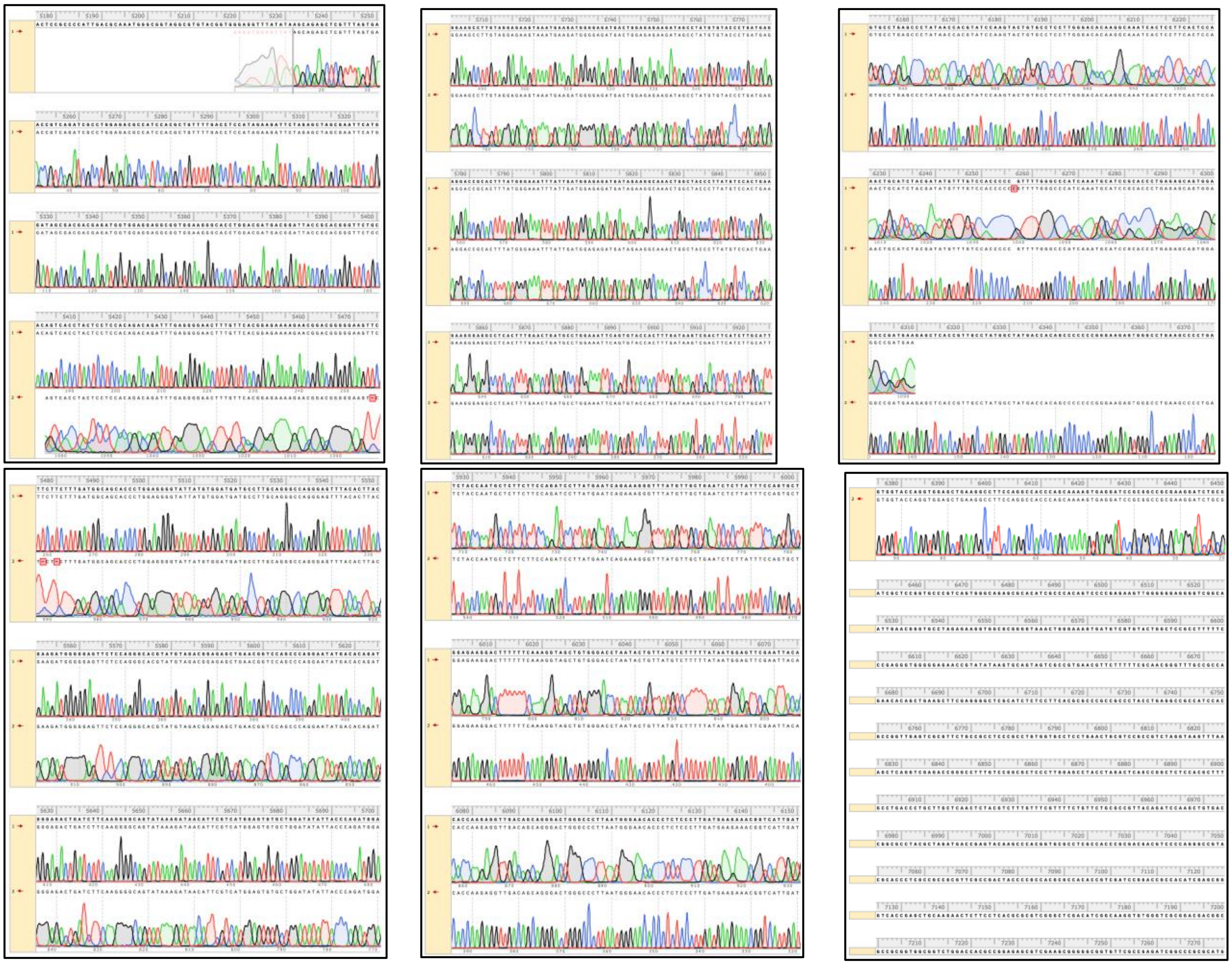

2. pACGFP1-LC3B-WT

(1) Plasmid map:

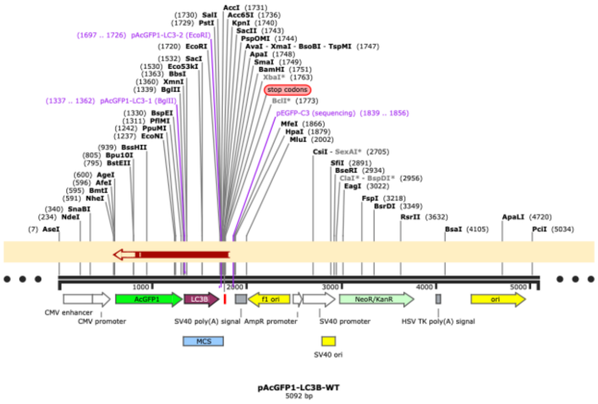

(2) Sequencing data:

Prime 1: pEGFP-C3

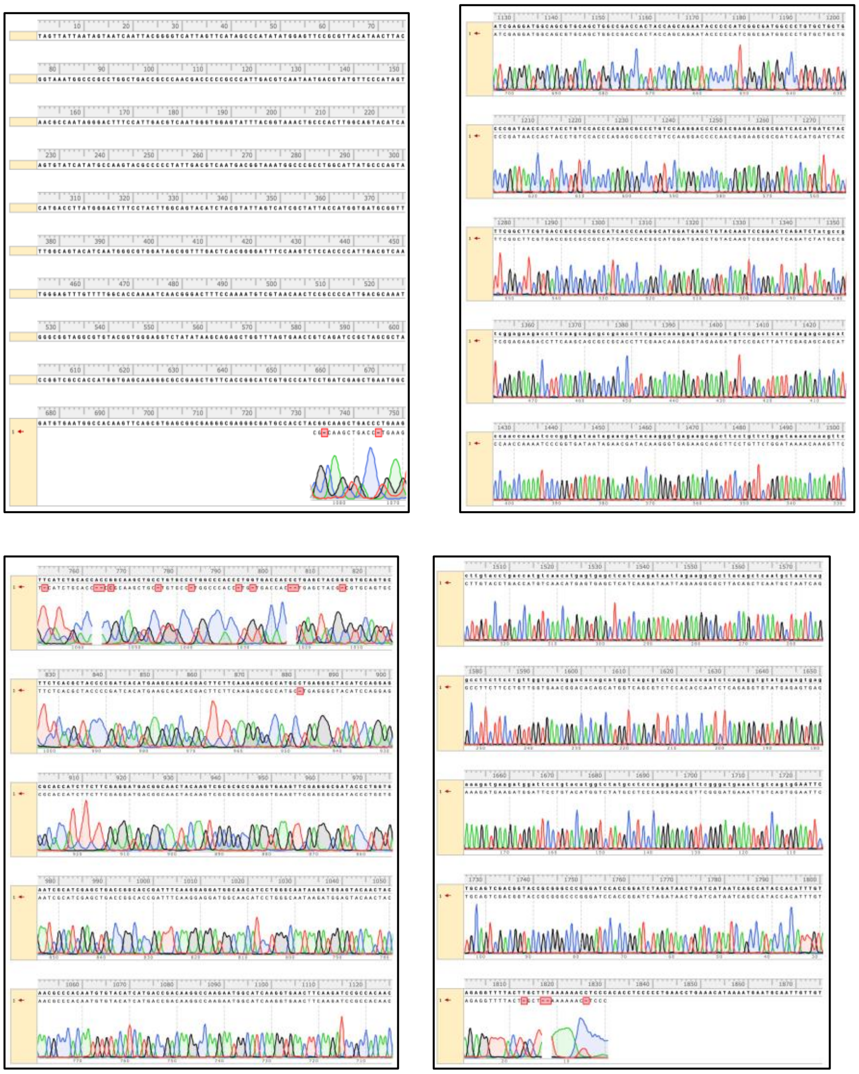

3. pACGFP1-LC3B-K51M

(1) Plasmid map:

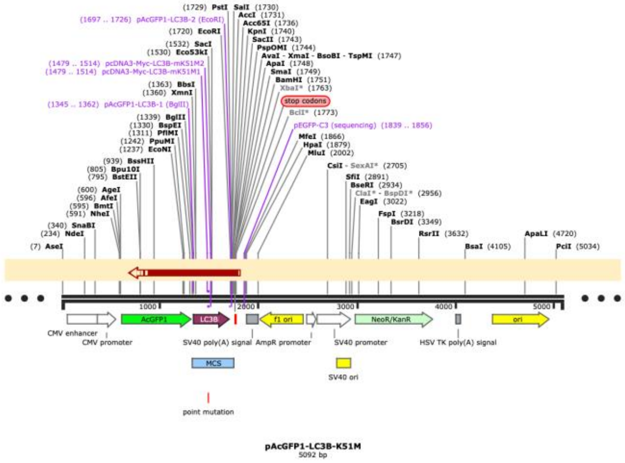

(2) Sequencing data:

Prime 1: pEGFP-C3

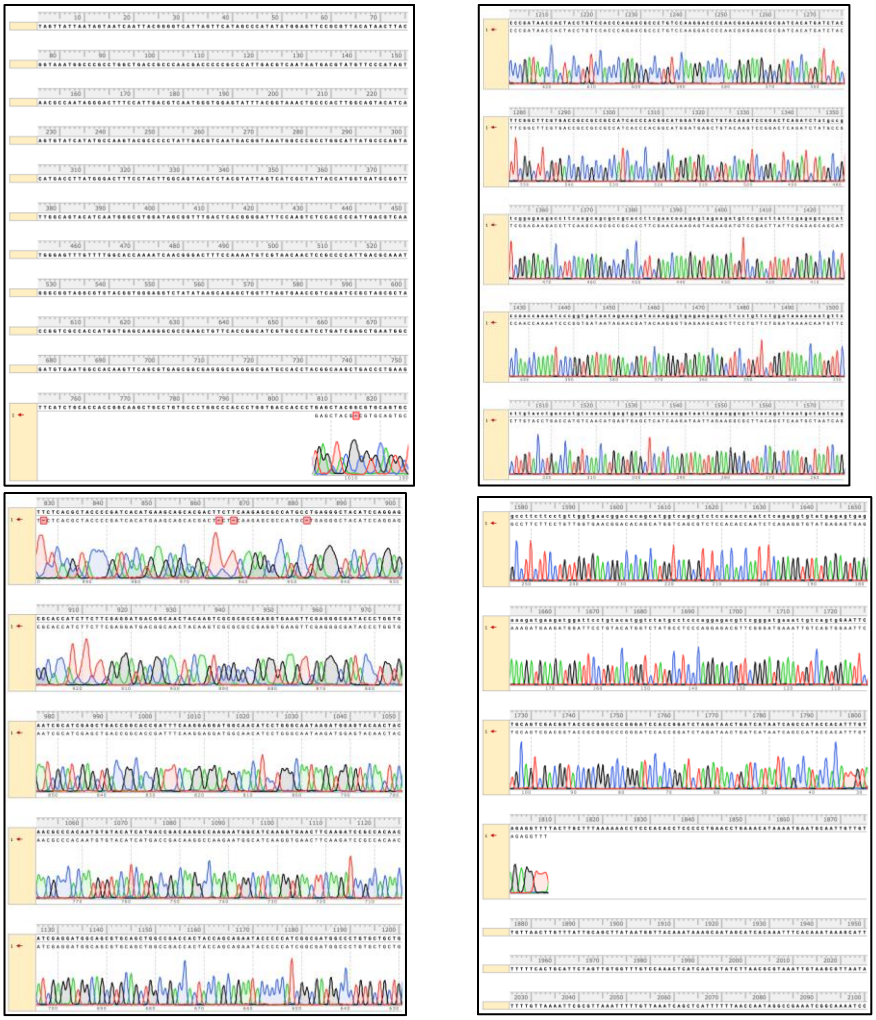

4. pcDNA3-MYC-LC3B-WT

(1) Plasmid map:

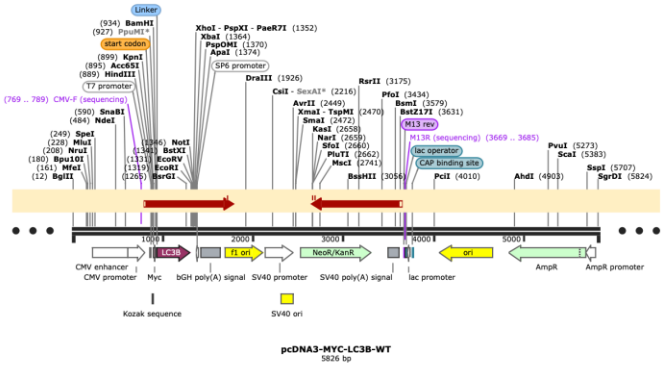

(2) Sequencing data:

Primer 1: CMV-F

Primer 2: M13R

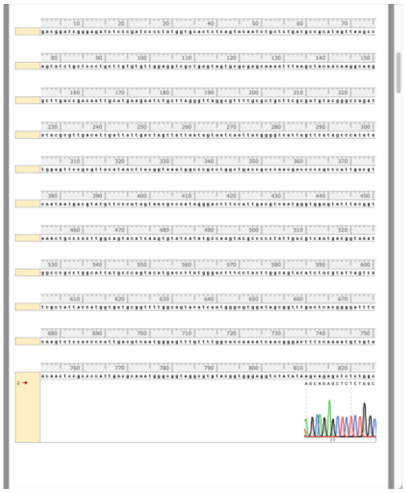

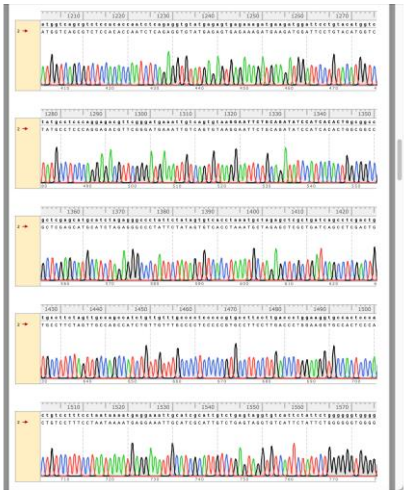

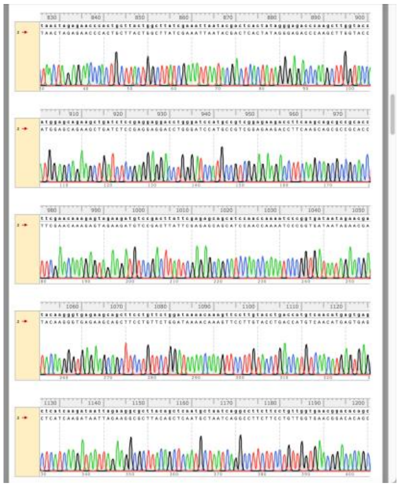

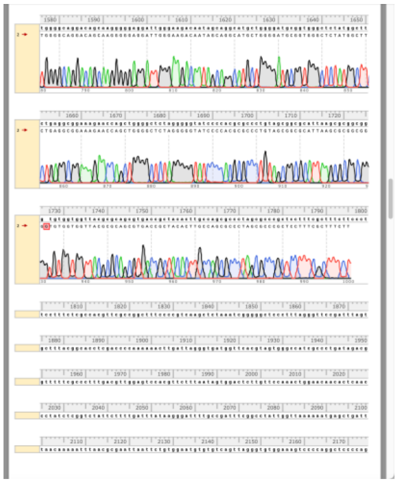

5. pcDNA3-MYC-LC3B-K51M

(1) Plasmid map:

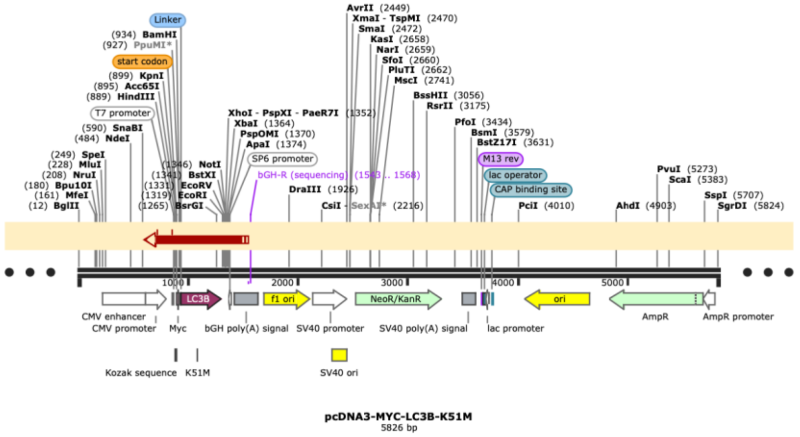

(2) Sequencing data:

Prime 1: bGH-R

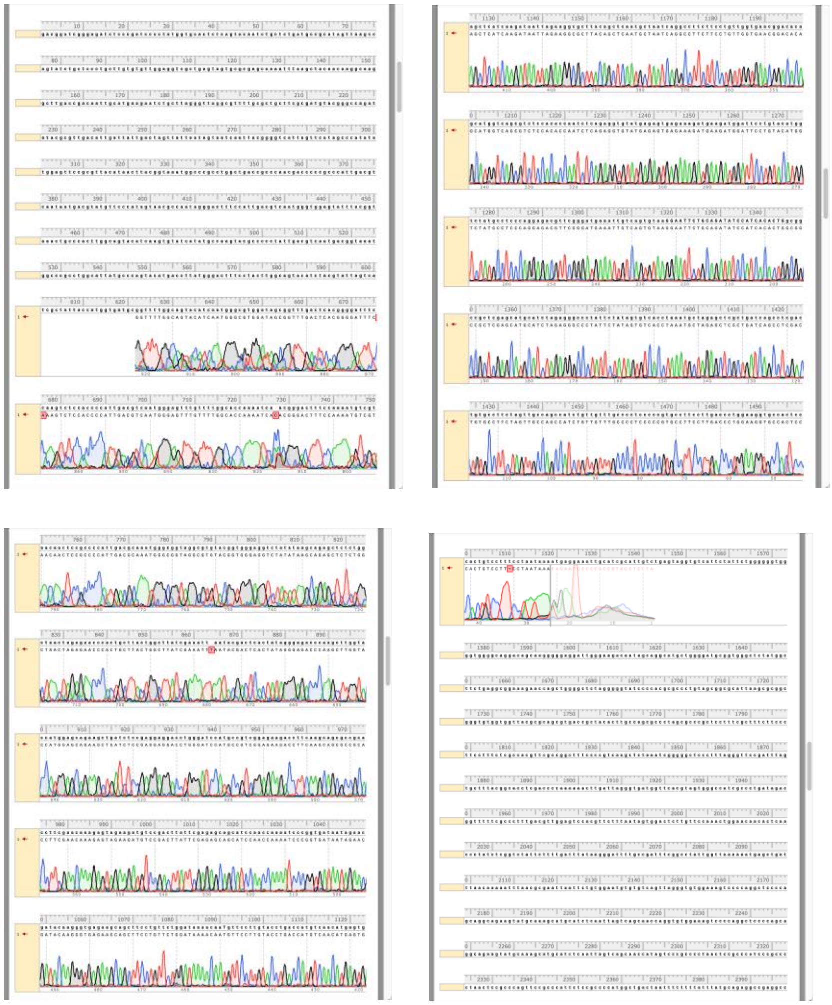

6. pLKO.1-shSETD7-1

(1) Plasmid map:

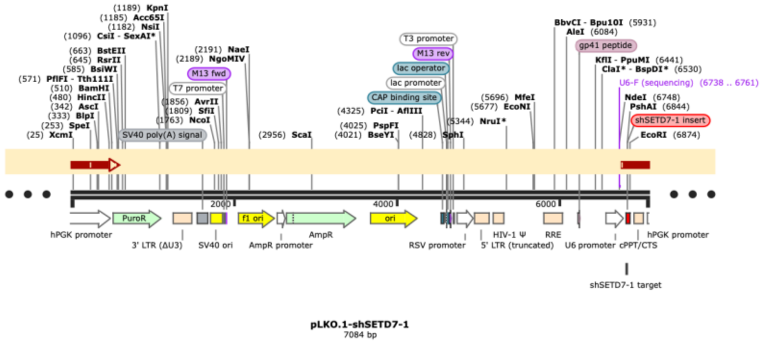

(2) Sequencing data:

Prime 1: U6-F

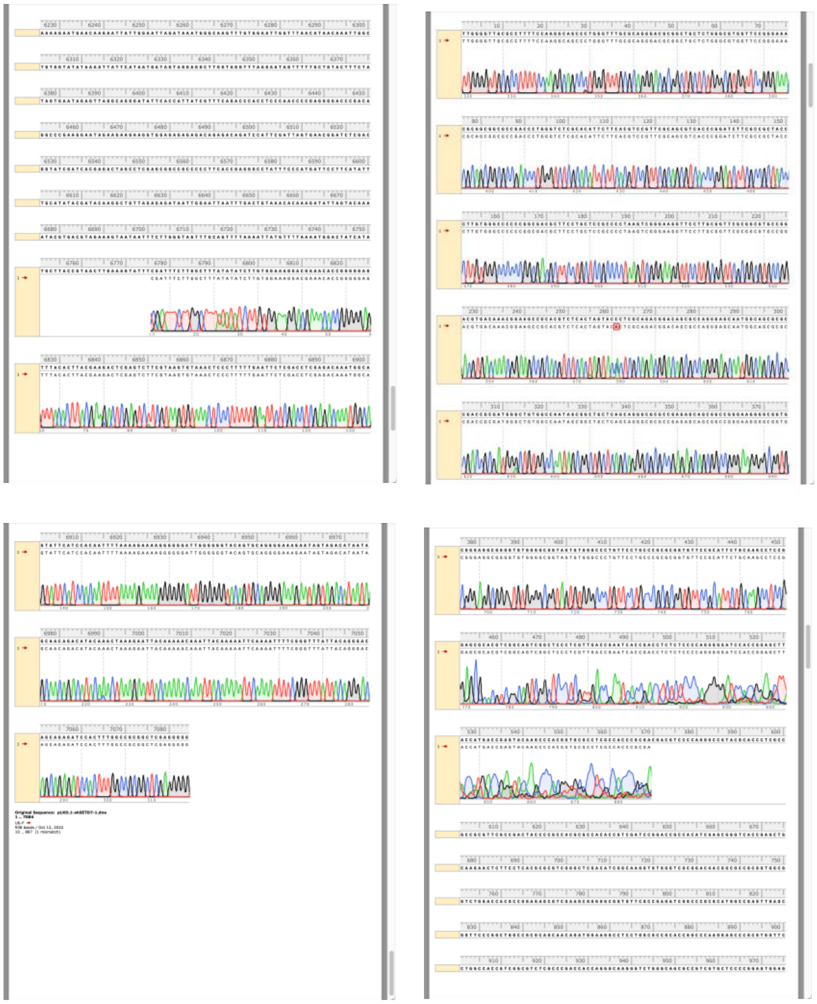

7. pLKO.1-shSETD7-2

(1) Plasmid map:

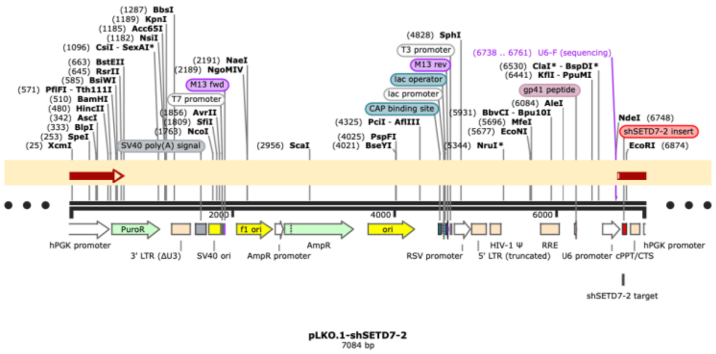

(2) Sequencing data:

Prime 1: U6-F

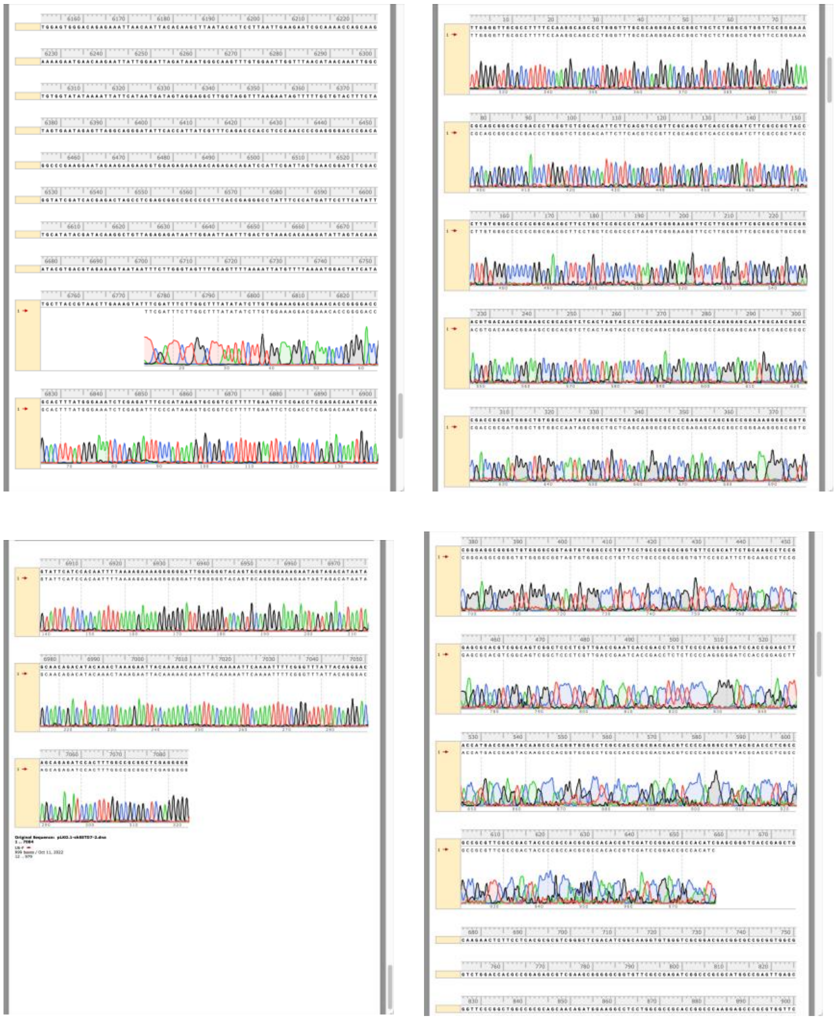

8. pLKO.1-shSETD7-3

(1) Plasmid map:

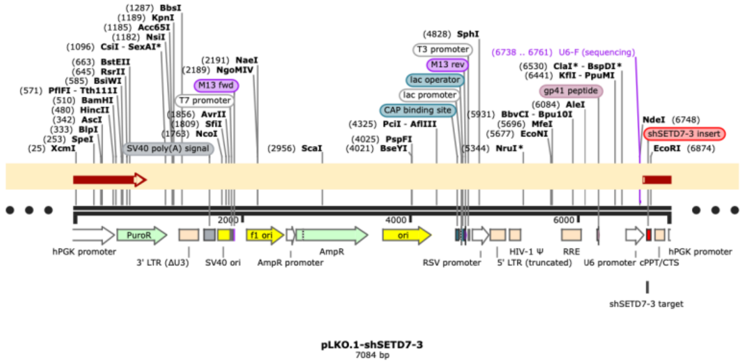

(2) Sequencing data:

Prime 1: U6-F

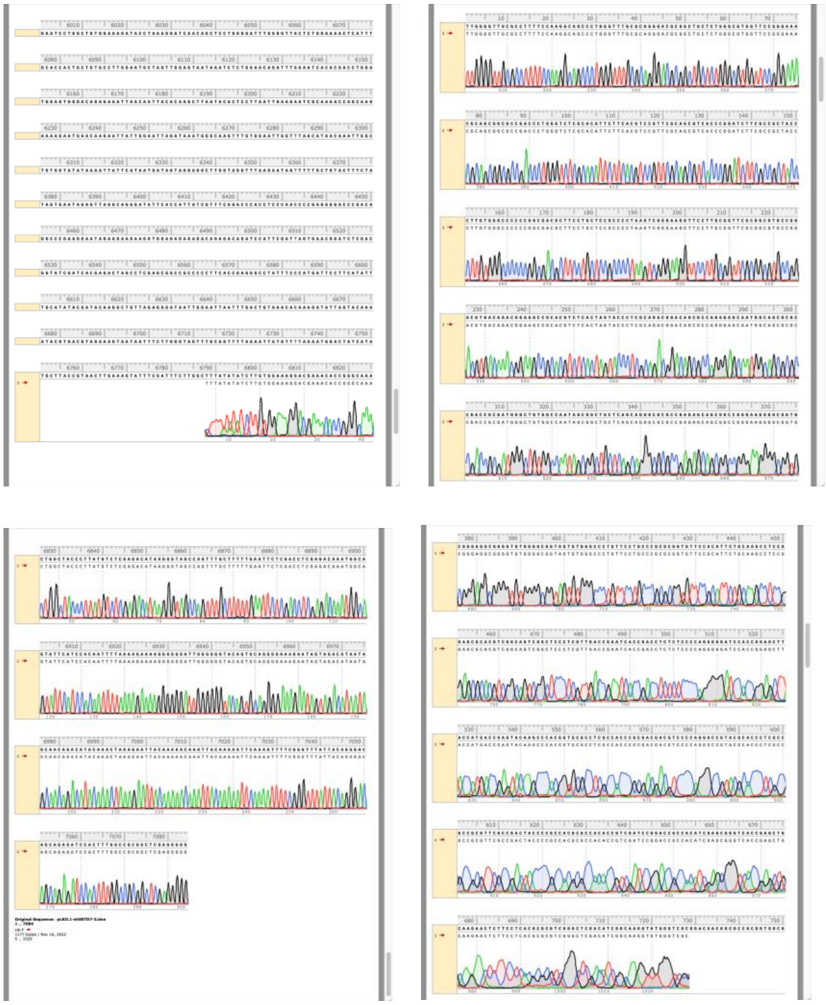

9. pCMV-Flag-ubiquitin

(1) Plasmid map:

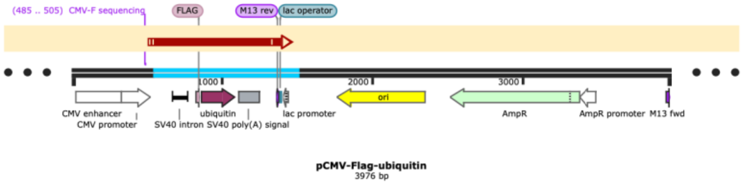

(2) Sequencing data:

Prime 1: CMV-F

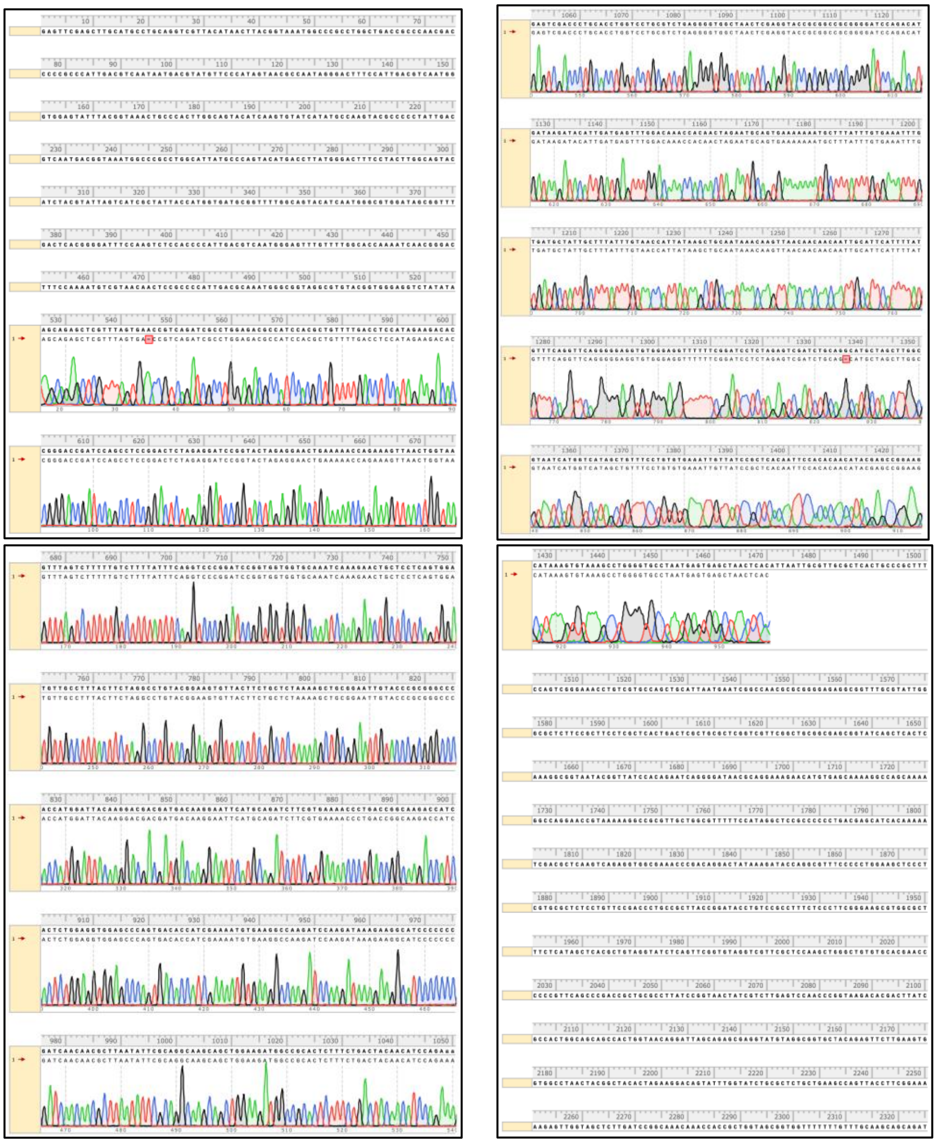

10. pCMV3-N-HA-SETD7

(1) Plasmid map:

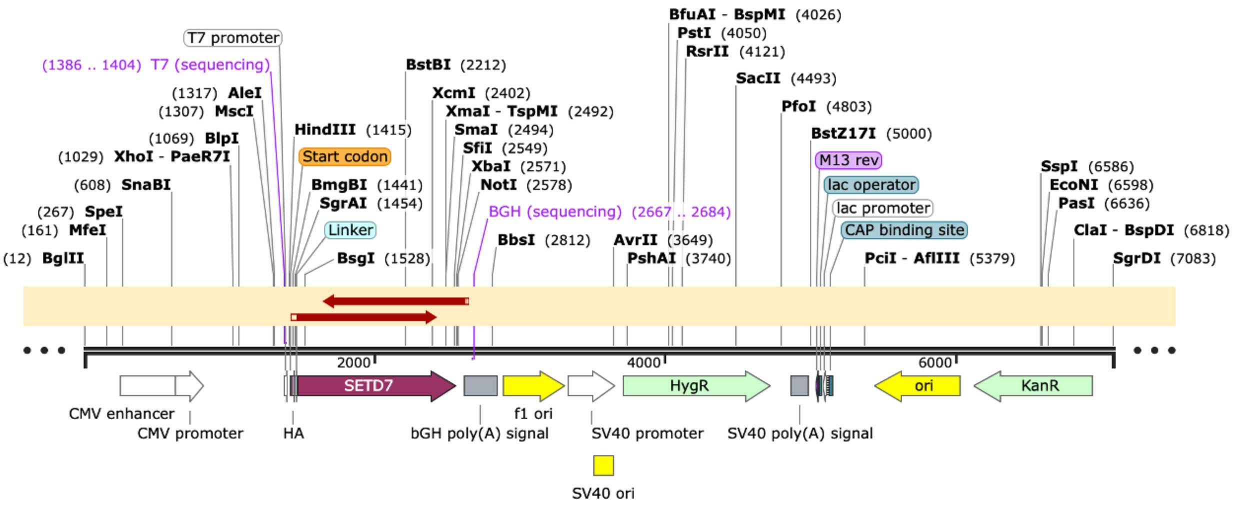

pCMV3-N-HA-SETD7
7085 bp

(2) sequencing data:

Prime 1: BGH

Prime 2: T7

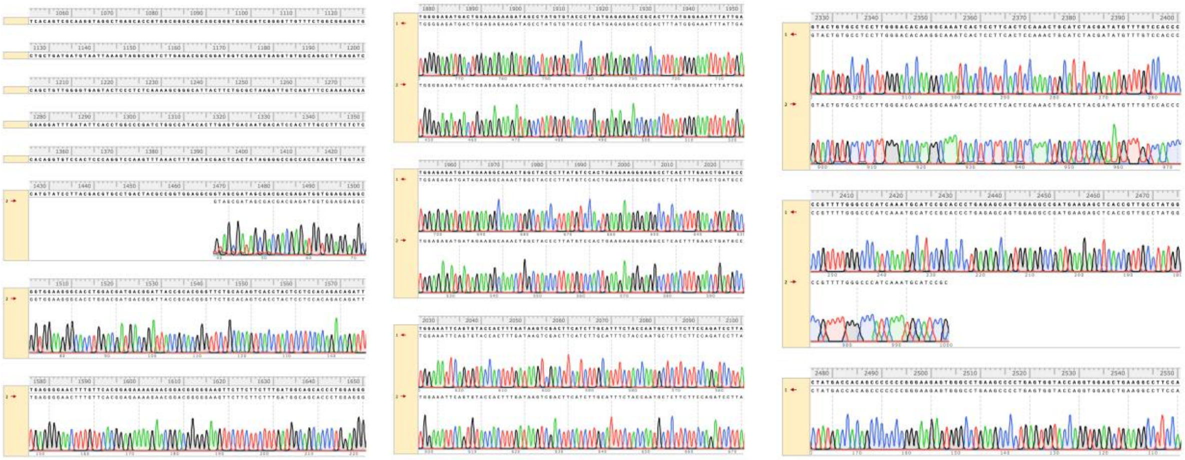

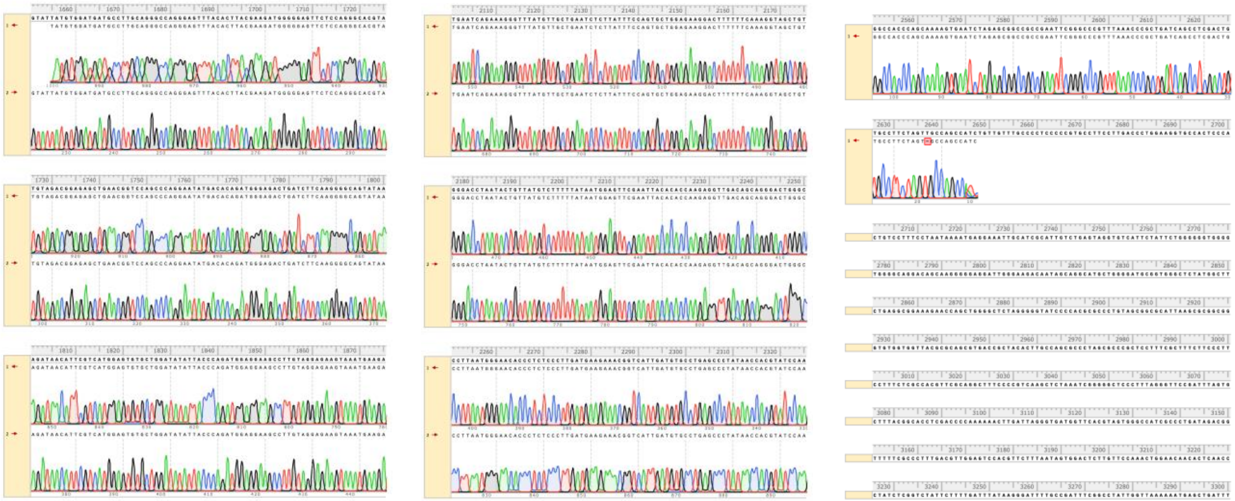

Supplement: Plasmid Sequencing Data [file mmc2.pdf]
