# Supplementary material for: SETD7 promotes LC3B methylation and degradation in ovarian cancer
Source: J Biol Chem. 2024 Dec 25;301(2):108134. doi: 10.1016/j.jbc.2024.108134 (PMC11791264; doi:10.1016/j.jbc.2024.108134)

Fig. 1A

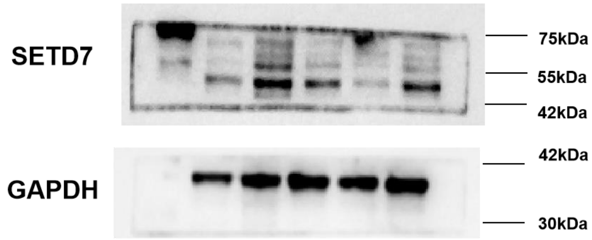

Fig. 1B

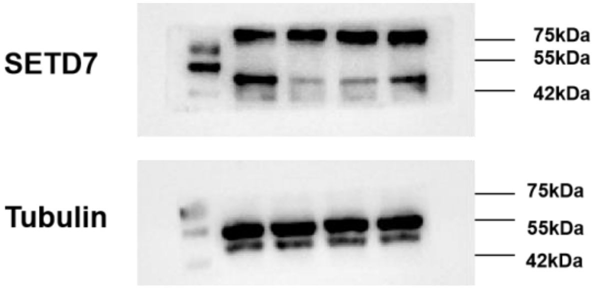

Fig. 1G

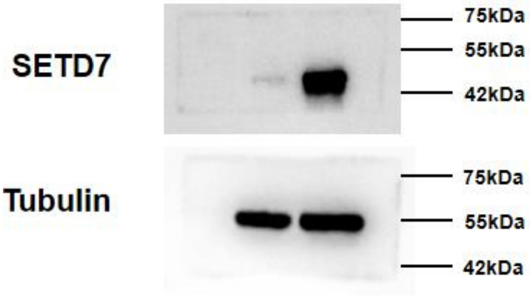

Fig. 1K

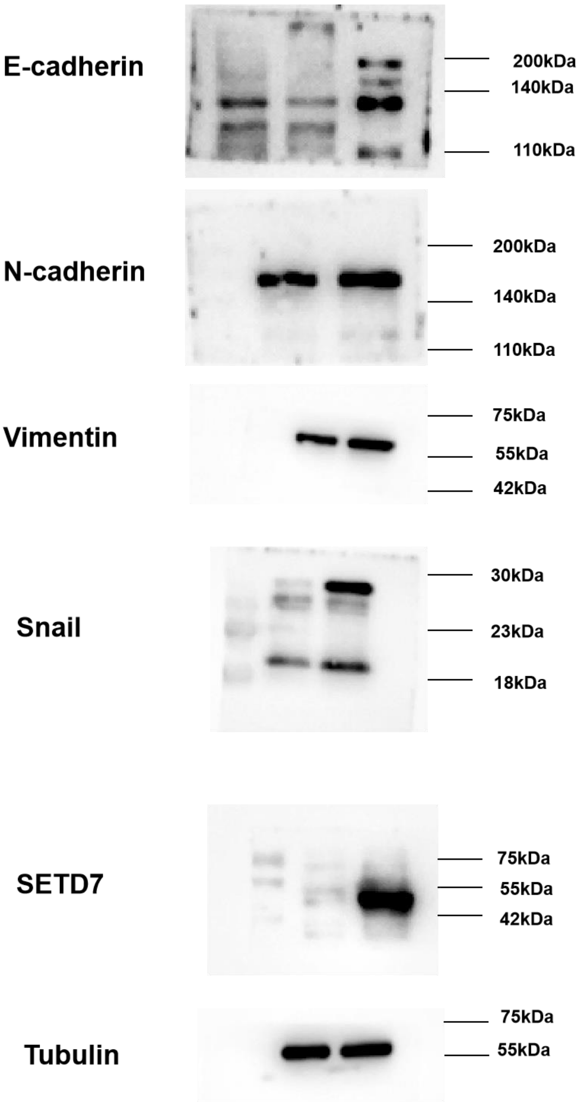

Fig. 2A

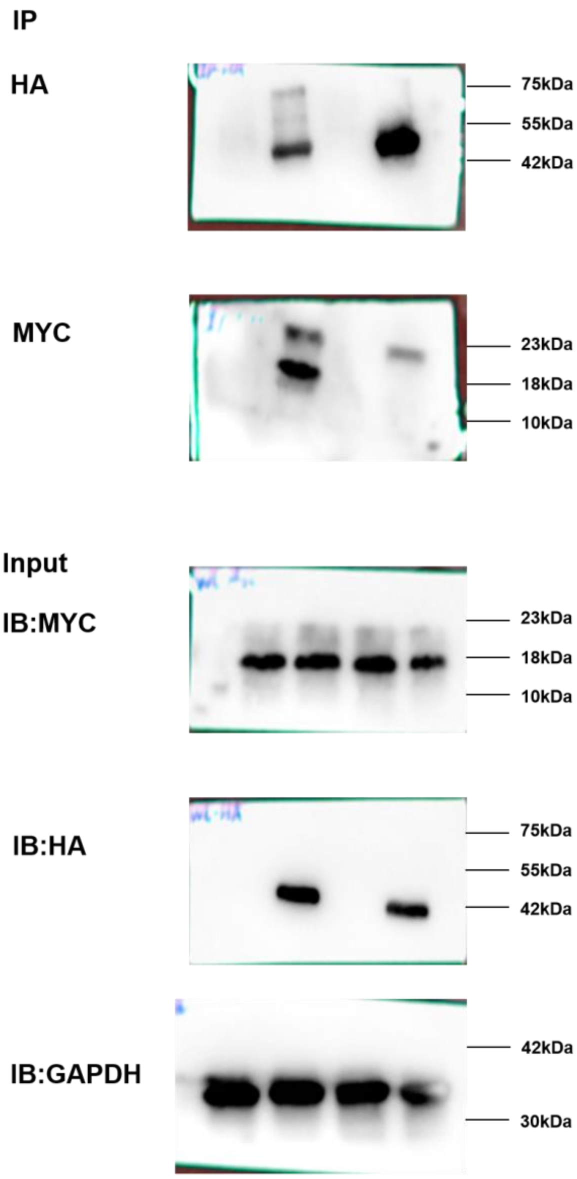
IP
HA
75kDa
55kDa
42kDa
MYC
23kDa
18kDa
10kDa
Input
IB:MYC
23kDa
18kDa
10kDa
IB:HA
75kDa
55kDa
42kDa
IB:GAPDH
42kDa
30kDa

Fig. 2B

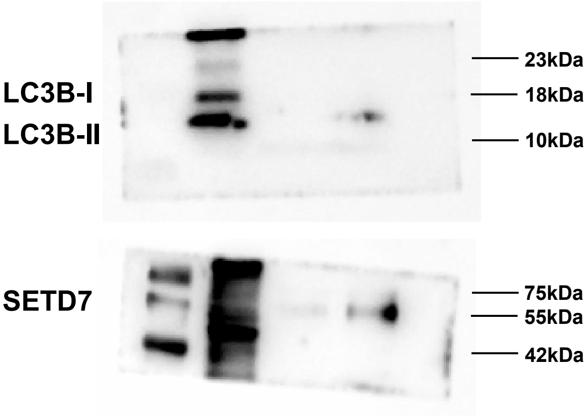
LC3B-I
LC3B-II
23kDa
18kDa
10kDa
SETD7
75kDa
55kDa
42kDa

Fig. 2C

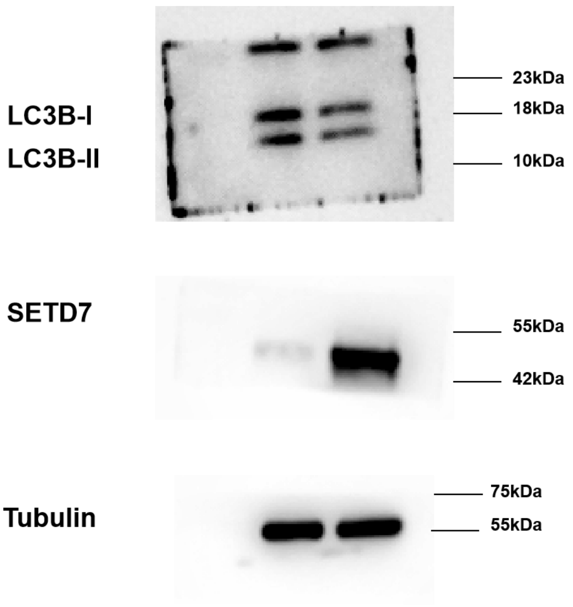
LC3B-I
LC3B-II
23kDa
18kDa
10kDa
SETD7
55kDa
42kDa
Tubulin
75kDa
55kDa

Fig. 2E

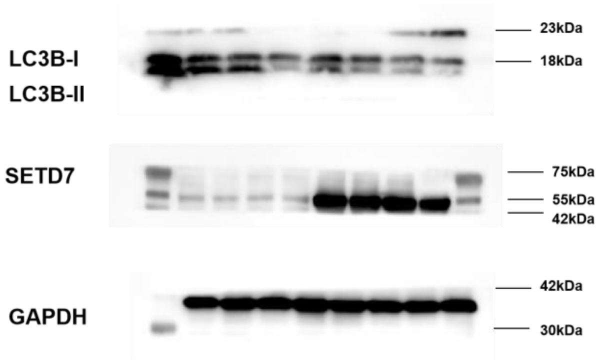
LC3B-I
LC3B-II
23kDa
18kDa
SETD7
75kDa
55kDa
42kDa
GAPDH
42kDa
30kDa

Fig. 2F

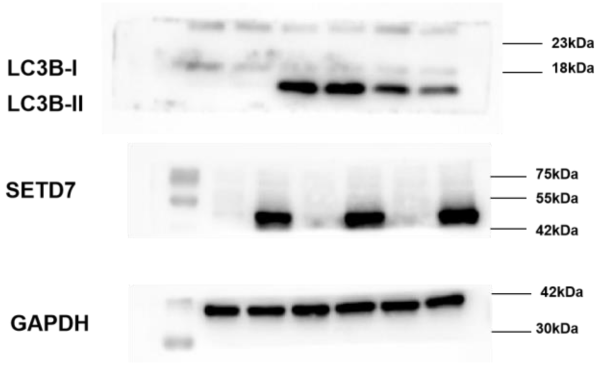
LC3B-I
LC3B-II
23kDa
18kDa
SETD7
75kDa
55kDa
42kDa
GAPDH
42kDa
30kDa

Fig. 2G

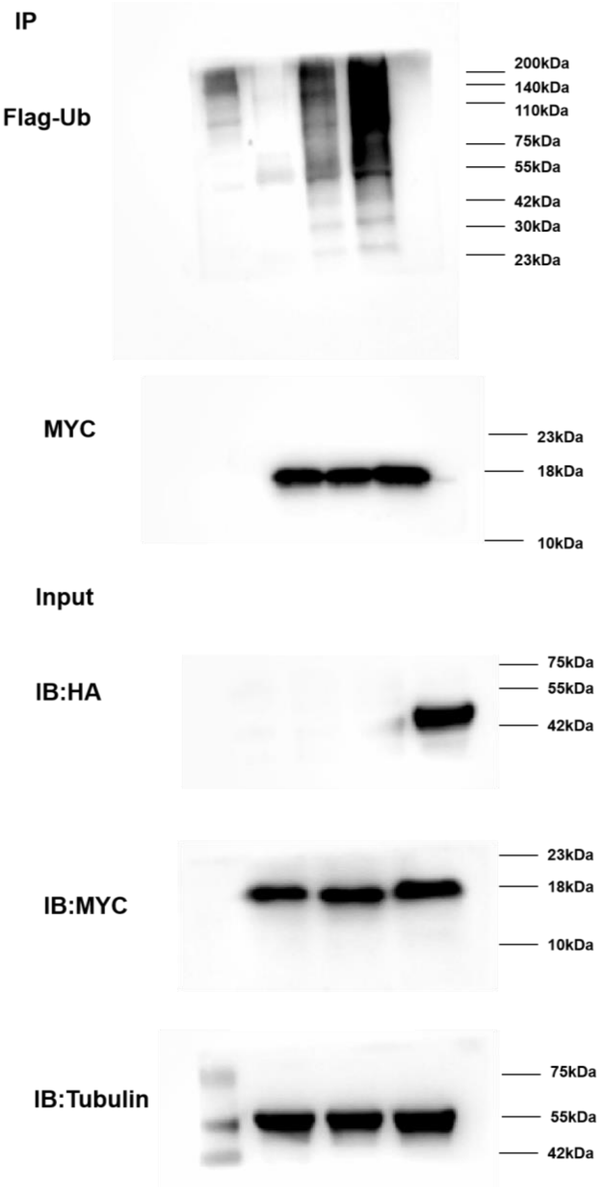
IP
Flag-Ub
200kDa
140kDa
110kDa
75kDa
55kDa
42kDa
30kDa
23kDa
MYC
23kDa
18kDa
10kDa
Input
IB:HA
75kDa
55kDa
42kDa
IB:MYC
23kDa
18kDa
10kDa
IB:Tubulin
75kDa
55kDa
42kDa

Fig. 2H

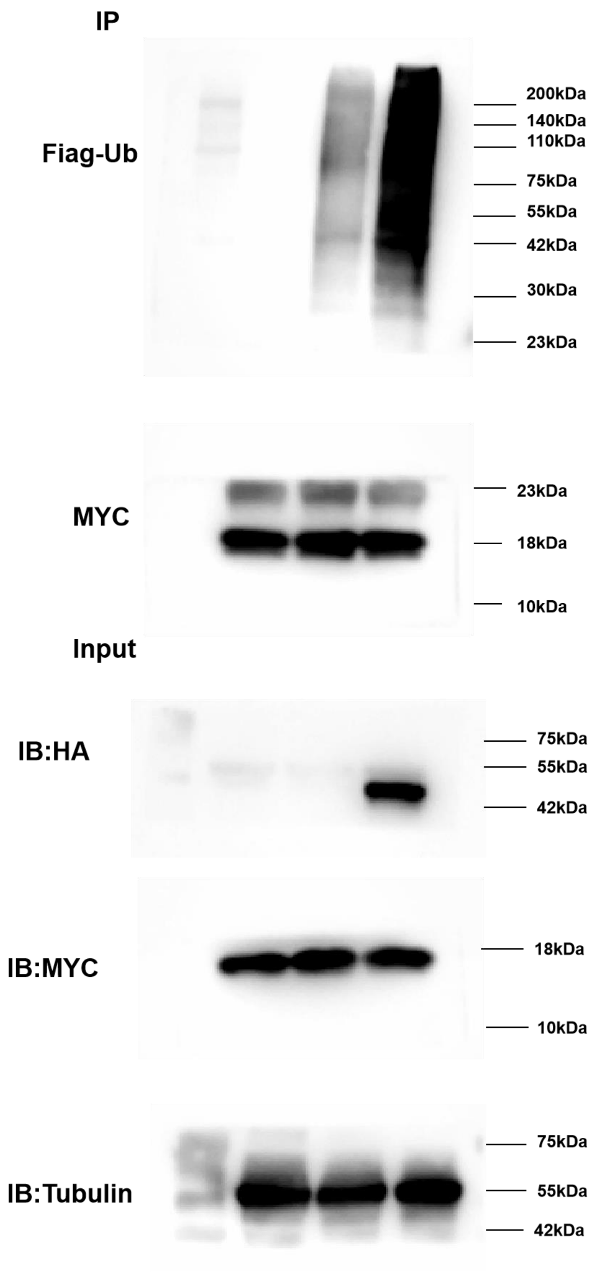
IP
Fiag-Ub
200kDa
140kDa
110kDa
75kDa
55kDa
42kDa
30kDa
23kDa
MYC
23kDa
18kDa
10kDa
Input
IB:HA
75kDa
55kDa
42kDa
IB:MYC
18kDa
10kDa
IB:Tubulin
75kDa
55kDa
42kDa

Fig. 3A

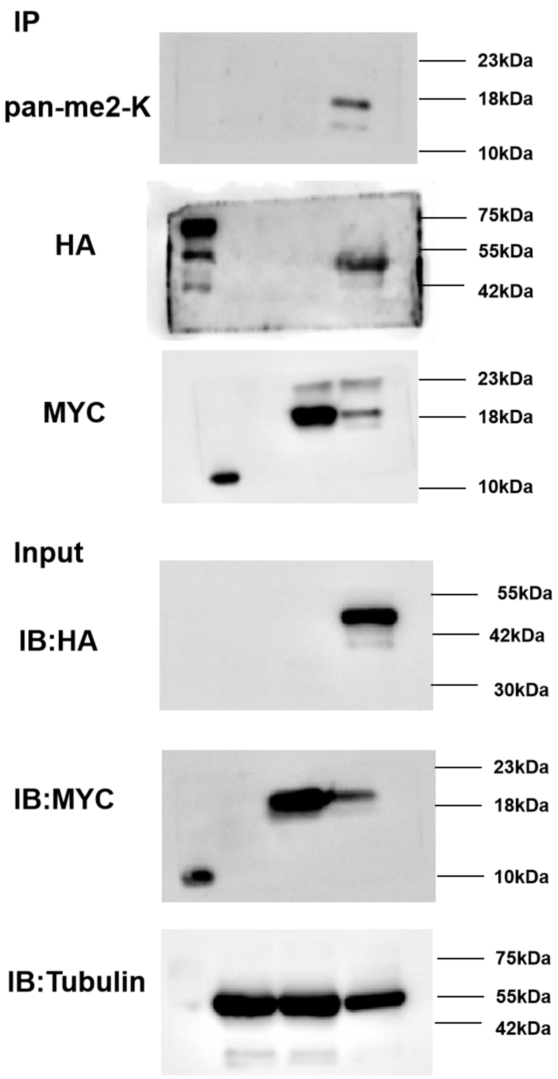
IP
pan-me2-K
23kDa
18kDa
10kDa
HA
75kDa
55kDa
42kDa
MYC
23kDa
18kDa
10kDa
Input
IB:HA
55kDa
42kDa
30kDa
IB:MYC
23kDa
18kDa
10kDa
IB:Tubulin
75kDa
55kDa
42kDa

Fig. 3B

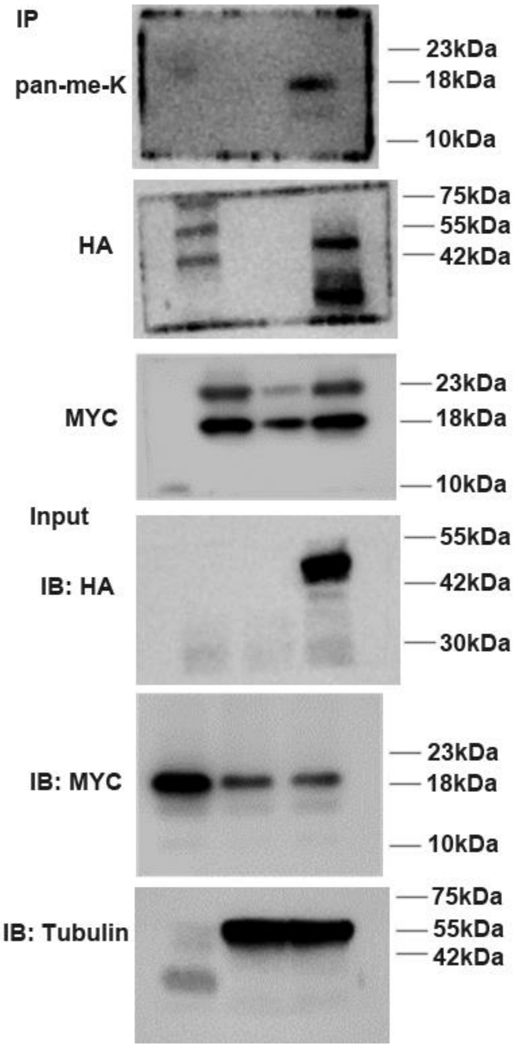
IP
pan-me-K
23kDa
18kDa
10kDa
HA
75kDa
55kDa
42kDa
MYC
23kDa
18kDa
10kDa
Input
IB: HA
55kDa
42kDa
30kDa
IB: MYC
23kDa
18kDa
10kDa
IB: Tubulin
75kDa
55kDa
42kDa

Fig. 3D

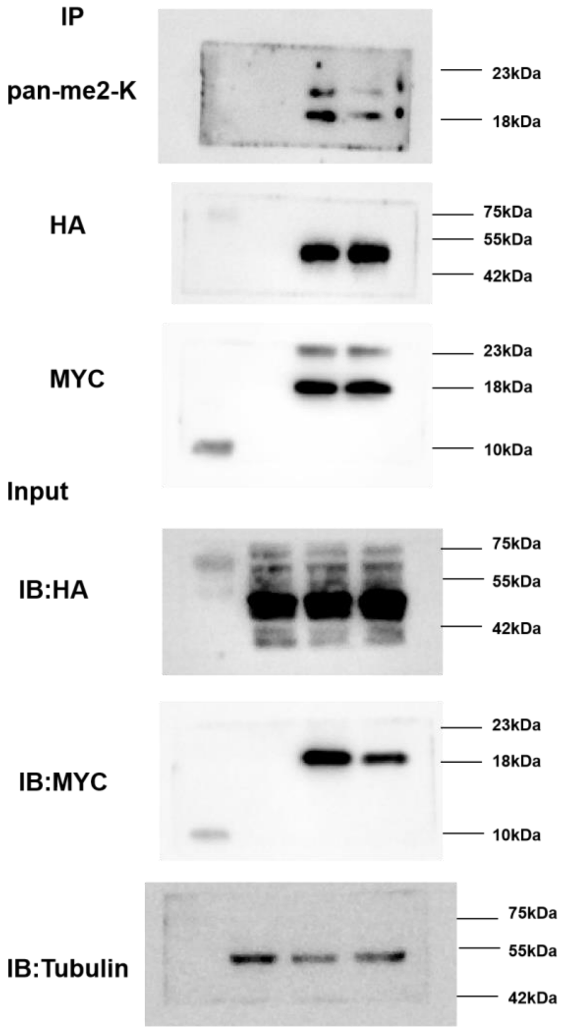
IP
pan-me2-K
23kDa
18kDa
HA
75kDa
55kDa
42kDa
MYC
23kDa
18kDa
10kDa
Input
IB:HA
75kDa
55kDa
42kDa
IB:MYC
23kDa
18kDa
10kDa
IB:Tubulin
75kDa
55kDa
42kDa

Fig. 3E

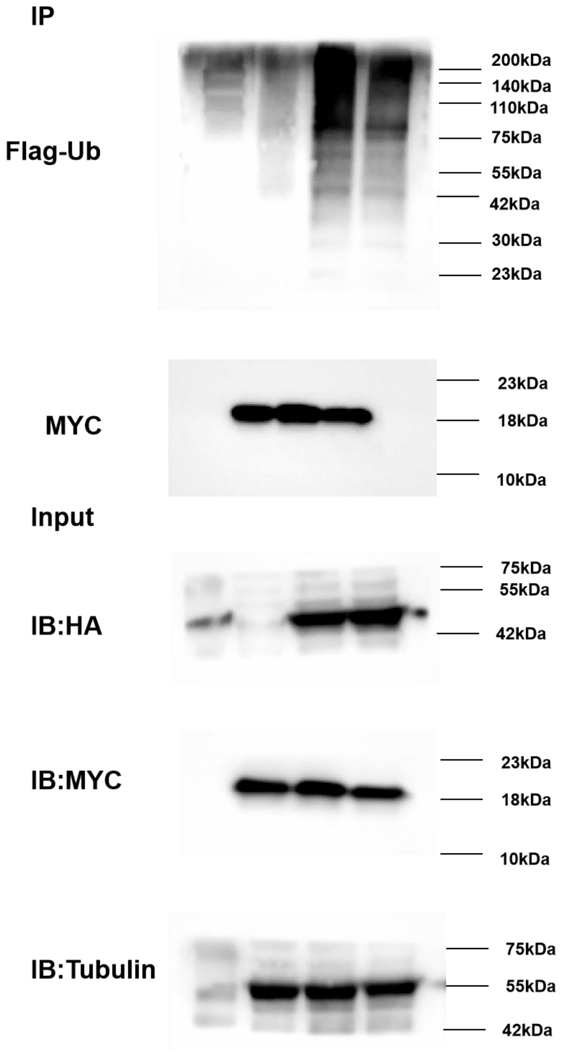
IP
Flag-Ub
200kDa
140kDa
110kDa
75kDa
55kDa
42kDa
30kDa
23kDa
MYC
23kDa
18kDa
10kDa
Input
IB:HA
75kDa
55kDa
42kDa
IB:MYC
23kDa
18kDa
10kDa
IB:Tubulin
75kDa
55kDa
42kDa

Fig. 3F

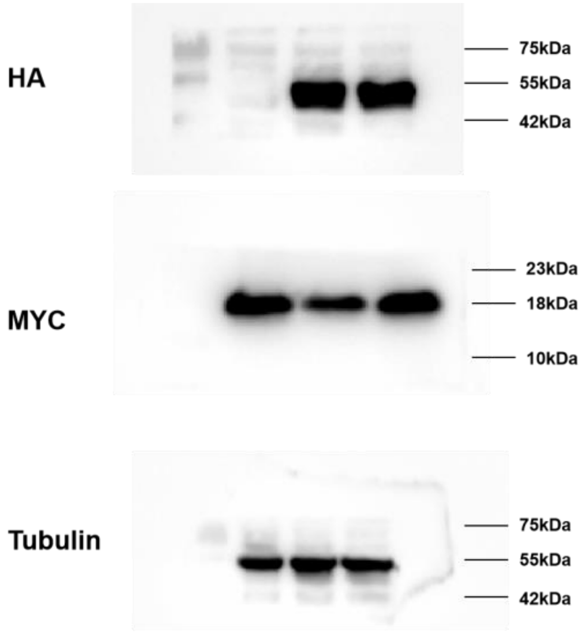
HA
75kDa
55kDa
42kDa
MYC
23kDa
18kDa
10kDa
Tubulin
75kDa
55kDa
42kDa

Fig. 4A

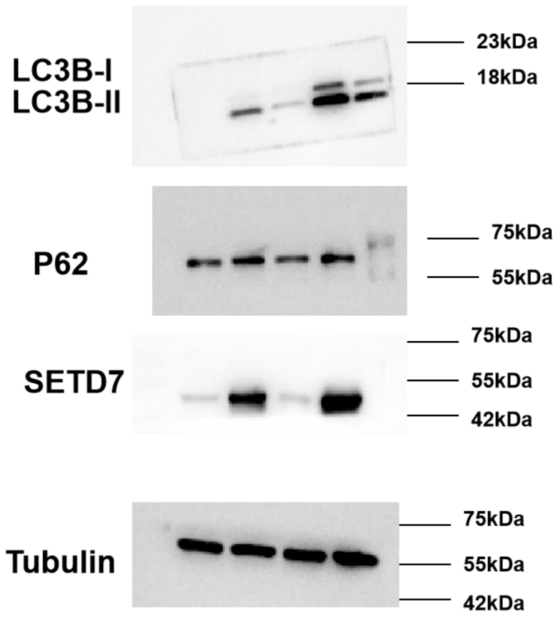

Fig. 4B

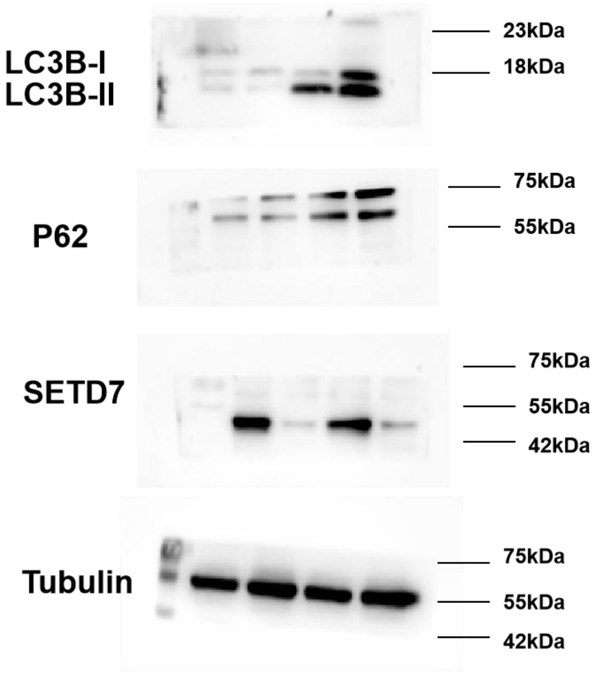

Fig. 4H

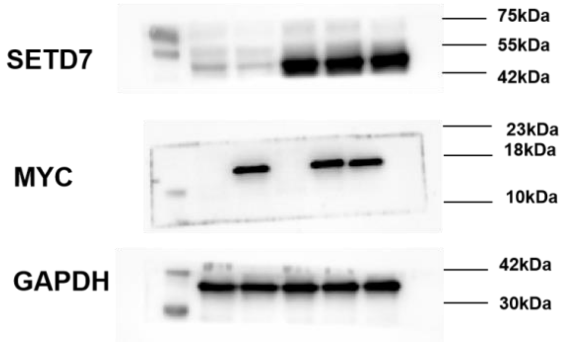

Supplement: Original Membranes for WB [file mmc3.pdf]
