# Supplementary material for: SETD7 promotes LC3B methylation and degradation in ovarian cancer
Source: J Biol Chem. 2024 Dec 25;301(2):108134. doi: 10.1016/j.jbc.2024.108134 (PMC11791264; doi:10.1016/j.jbc.2024.108134)
Supplement: Cell Line Authentication [file mmc4.pdf]

Cell Line Authentication Service

STR Genotype Testing Report

Submitting Institution: Shanghai Institute of Biochemistry and Cell Biology (SIBCB)

Sample Name: Cell Line

Testing Institution: Shanghai Biowing Applied Biotechnology Co., LTD

Report Date: 2022-06-24

Report Explanation

1. This report is only responsible for the samples submitted for inspection.
2. The inspection results and the name of the inspection unit on the inspection report shall not be used for advertising, evaluation, or commercial promotion without consent.
3. If you have any objections to this report, please submit them in writing within 15 days from the date of receipt. Requests submitted after the deadline will not be accepted.
4. Any alterations, additions, or deletions to the paper inspection report, or copies without the seal of the inspection unit, are invalid.

Sample Information

Sample Number:

Testing Sample Number	Company Number
SK-OV-3	20220616-10

Number of Samples: 1

Sample Name: Cell line

Testing Items: STR

Submitting Institution: Shanghai Institute of Biochemistry and Cell Biology (SIBCB)

Detection Method: DNA was extracted using Axygen's genome extraction kit, amplified using a 20-STR amplification protocol, and the STR loci and sex gene Amelogenin were detected on an ABI 3730XL analyzer.

Results

(1) Testing basic information

NO.	Multiple alleles	Matching cell lines	Cell bank	EV	Matching instructions
20220616-10	Yes	SK-OV-3 [SKOV-3]	DSMZ	1.00	Perfect match

Genotype test results

- Multi allelic genes refer to the phenomenon of genes with three or more alleles.
- The results of this genetic typing are valid.

(2) Description of each sample

- 20220616-10: The DNA typing of this cell line was matched perfectly in the cell line search. The DSMZ database shows that the cell name is SK-OV-3 [SKOV-3], and the cell number corresponds to HTB-77. Multiple alleles were found in this cell line.

EV	Cell No.	Cell name	Locus names									Figures
			D5S818	D13S317	D7S820	D16S539	VWA	TH01	AM	TPOX	CSF1PO	
	Query (Your Cell)		*11, 11*	*8, 11*	*13, 14*	*12, 12*	*17, 18*	*9, 9.3*	*X, X*	*8, 11*	*11, 11*	
1.00(36/36)	HTB-77	SK-OV-3 [SKOV-3]	11, 11	8, 11	13, 14	12, 12	17, 18	9, 9.3	X, X	8, 11	11, 11	-
0.72(26/36)	CCL-251	NCI-H716 [H716]	11, 11	8, 11	10, 11	11, 12	16, 17	6, 9.3	X, X	8, 11	11, 11	-
0.72(26/36)	CRL-1692	HISM	11, 11	8, 11	11, 12	12, 13	16, 17	9, 9.3	X, X	8, 11	11, 12	-

Note: The STR data of the submitted cell line was compared with that of the cell lines listed in ATCC, DSMZ, ExPASy, JCRB, and RIKEN databases. Cell lines that are not included in these databases will not be matched.

(3) Sample typing results

Genotyping results of STR and Amelogenin loci of the cell 20220616-10						
Loci	STR information for submitted sample			STR information for cell bank		
	Name of submitted sample: SK-OV-3			Name of cells in cell bank: SK-OV-3 [SKOV-3]		
	Allele1	Allele2	Allele3	Allele1	Allele2	Allele3
D5S818	11	11		11	11	
D13S317	8	11		8	11	
D7S820	13	14		13	14	
D16S539	12	12		12	12	
VWA	17	18		17	18	
TH01	9	9.3		9	9.3	
AMEL	X	X		X	X	
TPOX	8	11		8	11	
CSF1PO	11	11		11	11	
D12S391	22	23				
FGA	24	25				
D2S1338	18	23				
D21S11	30	31.2				
D18S51	16	17	18			
D8S1179	14	15				
D3S1358	14	14				
D6S1043	12	12				
PENTAE	5	13				
D19S433	14	14				
PENTAD	12	13				

Other Instructions

(1) Typing scheme and site distribution

	Scheme 1	Scheme 2	Scheme 3	Scheme 4
1	TH01	TPOX	D3S1358	AMEL
2	D12S391	VWA	D13S317	D5S818
3	D7S820	D8S1179	D6S1043	D2S1338
4	CSF1PO	PENTAD	D16S539	D21S11
5	FGA		D19S433	D18S51
6	PENTAE			

Typing scheme and loci

(2) STR database comparison

Our company uses DSMZ tools for cell line comparison, which includes 2455 cell line STR data from ATCC, DSMZ, ExPASy, JCRB, and RIKEN databases. If the cells to be tested are not included in the above cell bank or if they are newly established cell lines, they cannot be compared. Users need to compare their cell typing results with other databases on their own.

(3) References

[1] Authentication testing of HEK 293T and HeLa cell lines have been performed by Shanghai Biowi ng Applied Biotechnology Co.,Ltd via STR profiling. STR profiles match the standards recomme nded for HEK 293T and HeLa cell lines authentication.

[2] AGS, NCI-N87, HGC-27 and HEK293 were STR-authenticated on Dec. 8, 2015 by Shanghai Biowing Applied Biotechnology Co. LTD, Shanghai, China.

Experimenter: Jianan Zhang

Reviewer: Lijin Gong

Head: Yiqun Chen

Date of Issue：2022-06-24

Cell Line Authentication Service

STR Genotype Testing Report

Submitting Institution: Shanghai Institute of Biochemistry and Cell Biology (SIBCB)

Sample Name: Cell Line

Testing Institution: Shanghai Biowing Applied Biotechnology Co., LTD

Report Date: 2023-06-29

Report Explanation

1. This report is only responsible for the samples submitted for inspection.
2. The inspection results and the name of the inspection unit on the inspection report shall not be used for advertising, evaluation, or commercial promotion without consent.
3. If you have any objections to this report, please submit them in writing within 15 days from the date of receipt. Requests submitted after the deadline will not be accepted.
4. Any alterations, additions, or deletions to the paper inspection report, or copies without the seal of the inspection unit, are invalid.

Sample Information

Sample Number:

Testing Sample Number	Company Number
A2780	20230626-05

Number of Samples: 1

Sample Name: Cell line

Testing Items: STR

Submitting Institution: Shanghai Institute of Biochemistry and Cell Biology (SIBCB)

Detection Method: DNA was extracted using Axygen's genome extraction kit, amplified using a 20-STR amplification protocol, and the STR loci and sex gene Amelogenin were detected on an ABI 3730XL analyzer.

Results

(1) Testing basic information

NO.	Multiple alleles	Matching cell lines	Cell bank	EV	Matching instructions
20230626-05	Yes	A2780	ExPASy	0.89	Basic match

Genotype test results

- Multi allelic genes refer to the phenomenon of genes with three or more alleles.
- The results of this genetic typing are valid.

(2) Description of each sample

- 20230626-05: The DNA typing of this cell line found a basic match in the cell line search. The ExPASy database shows that the cell name is A2780 and the cell number corresponds to CVCL_0134. Multiple alleles were found in this cell line.

EV	Cell No.	Cell name	Locus names								
			D5S818	D13S317	D7S820	D16S539	VWA	TH01	AM	TPOX	CSF1PO
	Query (Your Cell)		10,13	12,13	10,10,	11,13	15,16	6,6	X,X	8,10	10,11
0.89(32/36)	CVCL_0134	A2780	['11', '12']	['12', '13']	['10', '10']	['11', '13']	['15', '16']	['6', '6']	['X', 'X']	['8', '10']	['10', '11']

Note: The STR data of the submitted cell line was compared with that of the cell lines listed in ATCC, DSMZ, ExPASy, JCRB, and RIKEN databases. Cell lines that are not included in these databases will not be matched.

(3) Sample typing results

Genotyping results of STR and Amelogenin loci of the cell 20230626-05						
Loci	STR information for submitted sample			STR information for cell bank		
	Name of submitted sample: A2780			Name of cells in cell bank: A2780		
	Allele1	Allele2	Allele3	Allele1	Allele2	Allele3
D5S818	10	13		11	12	
D13S317	12	13		12	13	
D7S820	10	10		10	10	
D16S539	11	13		11	13	
VWA	15	16		15	16	
TH01	6	6		6	6	
AMEL	X	X		X	X	
TPOX	8	10		8	10	
CSF1PO	10	11		10	11	
D12S391	19	20				
FGA	19	24				
D2S1338	21	22				
D21S11	28	29				
D18S51	17	17				
D8S1179	15	17				
D3S1358	14	16				
D6S1043	11	17				
PENTAE	10	13				
D19S433	12	12				
PENTAD	8	9				
D1S1656	12	13	15			

Other Instructions

(1) Typing scheme and site distribution

	Scheme 1	Scheme 2	Scheme 3	Scheme 4
1	D3S1358	D8S1179	D19S433	AMEL
2	VWA	D21S11	TH01	D1S1656
3	D7S820	D16S539	D13S317	D5S818
4	CSF1PO	D2S1338	TPOX	D12S391
5	PENTAE	PENTAD	D18S51	FGA
6			D6S1043	

Typing scheme and loci

(2) STR database comparison

Our company uses DSMZ tools for cell line comparison, which includes 2455 cell line STR data from ATCC, DSMZ, ExPASy, JCRB, and RIKEN databases. If the cells to be tested are not included in the above cell bank or if they are newly established cell lines, they cannot be compared. Users need to compare their cell typing results with other databases on their own.

(3) References

[1] Authentication testing of HEK 293T and HeLa cell lines have been performed by Shanghai Biowi ng Applied Biotechnology Co.,Ltd via STR profiling. STR profiles match the standards recomme nded for HEK 293T and HeLa cell lines authentication.

[2] AGS, NCI-N87, HGC-27 and HEK293 were STR-authenticated on Dec. 8, 2015 by Shanghai Biowing Applied Biotechnology Co. LTD, Shanghai, China.

Experimenter: Xiuchuan He

Reviewer: Chenqian Zhang

Head: Min Wang

Date of Issue：2023-06-29

Cell Line Authentication Service

STR Genotype Testing Report

Submitting Institution: Shanghai Institute of Biochemistry and Cell Biology (SIBCB)

Sample Name: Cell Line

Testing Institution: Shanghai Biowing Applied Biotechnology Co., LTD

Report Date: 2022-03-03

Report Explanation

1. This report is only responsible for the samples submitted for inspection.
2. The inspection results and the name of the inspection unit on the inspection report shall not be used for advertising, evaluation, or commercial promotion without consent.
3. If you have any objections to this report, please submit them in writing within 15 days from the date of receipt. Requests submitted after the deadline will not be accepted.
4. Any alterations, additions, or deletions to the paper inspection report, or copies without the seal of the inspection unit, are invalid.

Sample Information

Sample Number:

Testing Sample Number	Company Number
Hey a8	20220228-02

Number of Samples: 1

Sample Name: Cell line

Testing Items: STR

Submitting Institution: Shanghai Institute of Biochemistry and Cell Biology (SIBCB)

Detection Method: DNA was extracted using Axygen's genome extraction kit, amplified using a 20-STR amplification protocol, and the STR loci and sex gene Amelogenin were detected on an ABI 3730XL analyzer.

Results

(1) Testing basic information

NO.	Multiple alleles	Matching cell lines	Cell bank	EV	Matching instructions
20220228-02	No	HEY A8	ExPASy	1.00	Perfect match

Genotype test results

- Multi allelic genes refer to the phenomenon of genes with three or more alleles.
- The results of this genetic typing are valid.

(2) Description of each sample

- 20220228-02: The DNA typing of this cell line was matched perfectly in the cell line search. The ExPASy database shows that the cell name is HEY A8 and the cell number corresponds to CVCL_8878. No multiple alleles were found in this cell line during this test.

EV	Cell No.	Cell name	Locus names								
			D5S818	D13S317	D7S820	D16S539	VWA	TH01	AM	TPOX	CSF1PO
	Query (Your Cell)		11,12	11,11	12,12	8,12	16,17	8,9.3	X,X	11,11	10,11
1.0(36/36)	CVCL_8878	HEY A8	['11', '12']	['11', '11']	['12', '12']	['8', '12']	['16', '17']	['8', '9.3']	['X', 'X']	['11', '11']	['10', '11']

Note: The STR data of the submitted cell line was compared with that of the cell lines listed in ATCC, DSMZ, ExPASy, JCRB, and RIKEN databases. Cell lines that are not included in these databases will not be matched.

(3) Sample typing results

Genotyping results of STR and Amelogenin loci of the cell 20220228-02						
Loci	STR information for submitted sample			STR information for cell bank		
	Name of submitted sample: Hey a8			Name of cells in cell bank: HEY A8		
	Allele1	Allele2	Allele3	Allele1	Allele2	Allele3
D5S818	11	12		11	12	
D13S317	11	11		11	11	
D7S820	12	12		12	12	
D16S539	8	12		8	12	
VWA	16	17		16	17	
TH01	8	9.3		8	9.3	
AMEL	X	X		X	X	
TPOX	11	11		11	11	
CSF1PO	10	11		10	11	
D12S391	17	22				
FGA	20	21				
D2S1338	24	25				
D21S11	30	30				
D18S51	15	15				
D8S1179	13	13				
D3S1358	16	16				
D6S1043	11	12				
PENTAE	7	13				
D19S433	13	14				
PENTAD	9	13				
D1S1656	12	15				

Other Instructions

(1) Typing scheme and site distribution

	Scheme 1	Scheme 2	Scheme 3	Scheme 4
1	D3S1358	D8S1179	D19S433	AMEL
2	VWA	D21S11	TH01	D1S1656
3	D7S820	D16S539	D13S317	D5S818
4	CSF1PO	D2S1338	TPOX	D12S391
5	PENTAE	PENTAD	D18S51	FGA
6			D6S1043	

Typing scheme and loci

(2) STR database comparison

Our company uses DSMZ tools for cell line comparison, which includes 2455 cell line STR data from ATCC, DSMZ, ExPASy, JCRB, and RIKEN databases. If the cells to be tested are not included in the above cell bank or if they are newly established cell lines, they cannot be compared. Users need to compare their cell typing results with other databases on their own.

(3) References

[1] Authentication testing of HEK 293T and HeLa cell lines have been performed by Shanghai Biowing Applied Biotechnology Co.,Ltd via STR profiling. STR profiles match the standards recommended for HEK 293T and HeLa cell lines authentication.

[2] AGS, NCI-N87, HGC-27 and HEK293 were STR-authenticated on Dec. 8, 2015 by Shanghai Biowing Applied Biotechnology Co. LTD, Shanghai, China.

Experimenter: Jianan Zhang

Reviewer: Chenqian Zhang

Head: Kaiyue Chao

Date of Issue：2022-03-03

Cell Line Authentication Service

STR Genotype Testing Report

Submitting Institution: Shanghai Institute of Biochemistry and Cell Biology (SIBCB)

Sample Name: Cell Line

Testing Institution: Shanghai Biowing Applied Biotechnology Co., LTD

Report Date: 2023-06-29

Report Explanation

1. This report is only responsible for the samples submitted for inspection.
2. The inspection results and the name of the inspection unit on the inspection report shall not be used for advertising, evaluation, or commercial promotion without consent.
3. If you have any objections to this report, please submit them in writing within 15 days from the date of receipt. Requests submitted after the deadline will not be accepted.
4. Any alterations, additions, or deletions to the paper inspection report, or copies without the seal of the inspection unit, are invalid.

Sample Information

Sample Number:

Testing Sample Number	Company Number
ES-2	20230626-07

Number of Samples: 1

Sample Name: Cell line

Testing Items: STR

Submitting Institution: Shanghai Institute of Biochemistry and Cell Biology (SIBCB)

Detection Method: DNA was extracted using Axygen's genome extraction kit, amplified using a 20-STR amplification protocol, and the STR loci and sex gene Amelogenin were detected on an ABI 3730XL analyzer.

Results

(1) Testing basic information

NO.	Multiple alleles	Matching cell lines	Cell bank	EV	Matching instructions
20230626-07	No	ES-2	DSMZ	1.00	Perfect match

Genotype test results

- Multi allelic genes refer to the phenomenon of genes with three or more alleles.
- The results of this genetic typing are valid.

(2) Description of each sample

- 20230626-07: The DNA typing of this cell line was matched perfectly in the cell line search. The DSMZ database shows that the cell name is ES-2 and the cell number corresponds to CRL-1978. No multiple alleles were found in this cell line during this test.

EV	Cell No.	Cell name	Locus names								
			D5S818	D13S317	D7S820	D16S539	VWA	TH01	AM	TPOX	CSF1PO
	Query (Your Cell)		11,13	11,11	11,11	11,13	16,17	9.3,9.3	X,X	8,12	10,15
1.0(36/36)	CRL-1978	ES-2	['11', '13']	['11', '11']	['11', '11']	['11', '13']	['16', '17']	['9.3', '9.3']	['X', 'X']	['8', '12']	['10', '15']

Note: The STR data of the submitted cell line was compared with that of the cell lines listed in ATCC, DSMZ, ExPASy, JCRB, and RIKEN databases. Cell lines that are not included in these databases will not be matched.

(3) Sample typing results

Genotyping results of STR and Amelogenin loci of the cell 20230626-07						
Loci	STR information for submitted sample			STR information for cell bank		
	Name of submitted sample: ES-2			Name of cells in cell bank: ES-2		
	Allele1	Allele2	Allele3	Allele1	Allele2	Allele3
D5S818	11	13		11	13	
D13S317	11	11		11	11	
D7S820	11	11		11	11	
D16S539	11	13		11	13	
VWA	16	17		16	17	
TH01	9.3	9.3		9.3	9.3	
AMEL	X	X		X	X	
TPOX	8	12		8	12	
CSF1PO	10	15		10	15	
D12S391	20	21				
FGA	21	21				
D2S1338	17	23				
D21S11	32.2	33.2				
D18S51	13	15				
D8S1179	14	14				
D3S1358	15	18				
D6S1043	11	12				
PENTAE	13	16				
D19S433	15	15.2				
PENTAD	8	13				
D1S1656	13	16				

Other Instructions

(1) Typing scheme and site distribution

	Scheme 1	Scheme 2	Scheme 3	Scheme 4
1	D3S1358	D8S1179	D19S433	AMEL
2	VWA	D21S11	TH01	D1S1656
3	D7S820	D16S539	D13S317	D5S818
4	CSF1PO	D2S1338	TPOX	D12S391
5	PENTAE	PENTAD	D18S51	FGA
6			D6S1043	

Typing scheme and loci

(2) STR database comparison

Our company uses DSMZ tools for cell line comparison, which includes 2455 cell line STR data from ATCC, DSMZ, ExPASy, JCRB, and RIKEN databases. If the cells to be tested are not included in the above cell bank or if they are newly established cell lines, they cannot be compared. Users need to compare their cell typing results with other databases on their own.

(3) References

[1] Authentication testing of HEK 293T and HeLa cell lines have been performed by Shanghai Biowing Applied Biotechnology Co.,Ltd via STR profiling. STR profiles match the standards recommended for HEK 293T and HeLa cell lines authentication.

[2] AGS, NCI-N87, HGC-27 and HEK293 were STR-authenticated on Dec. 8, 2015 by Shanghai Biowing Applied Biotechnology Co. LTD, Shanghai, China.

Experimenter: Xiuchuan He

Reviewer: Chenqian Zhang

Head: Min Wang

Date of Issue：2023-06-29

Cell Line Authentication Service

STR Genotype Testing Report

Submitting Institution: Shanghai Institute of Biochemistry and Cell Biology (SIBCB)

Sample Name: Cell Line

Testing Institution: Shanghai Biowing Applied Biotechnology Co., LTD

Report Date: 2022-06-15

Report Explanation

1. This report is only responsible for the samples submitted for inspection.
2. The inspection results and the name of the inspection unit on the inspection report shall not be used for advertising, evaluation, or commercial promotion without consent.
3. If you have any objections to this report, please submit them in writing within 15 days from the date of receipt. Requests submitted after the deadline will not be accepted.
4. Any alterations, additions, or deletions to the paper inspection report, or copies without the seal of the inspection unit, are invalid.

Sample Information

Sample Number:

Testing Sample Number	Company Number
KGN	20220608-03

Number of Samples: 1

Sample Name: Cell line

Testing Items: STR

Submitting Institution: Shanghai Institute of Biochemistry and Cell Biology (SIBCB)

Detection Method: DNA was extracted using Axygen's genome extraction kit, amplified using a 20-STR amplification protocol, and the STR loci and sex gene Amelogenin were detected on an ABI 3730XL analyzer.

Results

(1) Testing basic information

NO.	Multiple alleles	Matching cell lines	Cell bank	EV	Matching instructions
20220608-03	Yes	KGN	DSMZ	1.00	Perfect match

Genotype test results

- Multi allelic genes refer to the phenomenon of genes with three or more alleles.
- The results of this genetic typing are valid.

(2) Description of each sample

- 20220608-03: The DNA typing of this cell line was matched perfectly in the cell line search. The DSMZ database shows that the cell name is KGN and the cell number corresponds to RCB1154. Multiple alleles were found in this cell line during this test.

EV	Cell No.	Cell name	Locus names									Figures
			D5S818	D13S317	D7S820	D16S539	VWA	TH01	AM	TPOX	CSF1PO	
	Query (Your Cell)		13,13	9,9	11,11	10,12	17,17	9,9	X,X	8,9	10,12	
1.00(36/36)	RCB1154	KGN	13,13	9,9	11,11	10,12	17,17	9,9	X,X	8,9	10,12	-
0.78(28/36)	CRL-7476	Hs 739.Sk	13,13	8,9	11,11	9,12	14,18	9,9	X,X	8,9	10,12	-
0.78(28/36)	CRL-7477	Hs 739.T	13,13	8,9	11,11	9,12	14,18	9,9	X,X	8,9	10,12	-
0.67(24/36)	645	SHI-1	12,13	9,9	11,12	9,10	17,17	6,7	X,Y	8,9	10,12	-
0.67(24/36)	JCRB1019	RERF-LC-Sq1	13,13	9,9	11,12	9,11	17,17	9,9	X,X	8,11	9,11	-

Note: The STR data of the submitted cell line was compared with that of the cell lines listed in ATCC, DSMZ, ExPASy, JCRB, and RIKEN databases. Cell lines that are not included in these databases will not be matched.

(3) Sample typing results

Genotyping results of STR and Amelogenin loci of the cell 20220608-03						
Loci	STR information for submitted sample			STR information for cell bank		
	Name of submitted sample: KGN			Name of cells in cell bank: KGN		
	Allele1	Allele2	Allele3	Allele1	Allele2	Allele3
D5S818	13	13		13	13	
D13S317	9	9		9	9	
D7S820	11	11		11	11	
D16S539	10	12		10	12	
VWA	17	17		17	17	
TH01	9	9		9	9	
AMEL	X	X		X	X	
TPOX	8	9		8	9	
CSF1PO	10	12		10	12	
D12S391	19	21				
FGA	25	26				
D2S1338	25	25				
D21S11	30	32.2				
D18S51	12	14				
D8S1179	13	14				
D3S1358	14	15	17			
D6S1043	13	20				
PENTAE	12	21				
D19S433	13	15.2				
PENTAD	10	13				
D1S1656	15	17				

Other Instructions

(1) Typing scheme and site distribution

	Scheme 1	Scheme 2	Scheme 3	Scheme 4
1	D3S1358	D8S1179	D19S433	AMEL
2	VWA	D21S11	TH01	D1S1656
3	D7S820	D16S539	D13S317	D5S818
4	CSF1PO	D2S1338	TPOX	D12S391
5	PENTAE	PENTAD	D18S51	FGA
6			D6S1043	

Typing scheme and loci

(2) STR database comparison

Our company uses DSMZ tools for cell line comparison, which includes 2455 cell line STR data from ATCC, DSMZ, ExPASy, JCRB, and RIKEN databases. If the cells to be tested are not included in the above cell bank or if they are newly established cell lines, they cannot be compared. Users need to compare their cell typing results with other databases on their own.

(3) References

[1] Authentication testing of HEK 293T and HeLa cell lines have been performed by Shanghai Biowing Applied Biotechnology Co.,Ltd via STR profiling. STR profiles match the standards recommended for HEK 293T and HeLa cell lines authentication.

[2] AGS, NCI-N87, HGC-27 and HEK293 were STR-authenticated on Dec. 8, 2015 by Shanghai Biowing Applied Biotechnology Co. LTD, Shanghai, China.

Experimenter: Jianan Zhang

Reviewer: Ning Qian

Head: Yang Bai

Date of Issue：2022-06-15

Cell Line Authentication Service

STR Genotype Testing Report

Submitting Institution: Shanghai Institute of Biochemistry and Cell Biology (SIBCB)

Sample Name: Cell Line

Testing Institution: Shanghai Biowing Applied Biotechnology Co., LTD

Report Date: 2022-03-08

Report Explanation

1. This report is only responsible for the samples submitted for inspection.
2. The inspection results and the name of the inspection unit on the inspection report shall not be used for advertising, evaluation, or commercial promotion without consent.
3. If you have any objections to this report, please submit them in writing within 15 days from the date of receipt. Requests submitted after the deadline will not be accepted.
4. Any alterations, additions, or deletions to the paper inspection report, or copies without the seal of the inspection unit, are invalid.

Sample Information

Sample Number:

Testing Sample Number	Company Number
HEK-293T	20220301-02

Number of Samples: 1

Sample Name: Cell line

Testing Items: STR

Submitting Institution: Shanghai Institute of Biochemistry and Cell Biology (SIBCB)

Detection Method: DNA was extracted using Axygen's genome extraction kit, amplified using a 20-STR amplification protocol, and the STR loci and sex gene Amelogenin were detected on an ABI 3730XL analyzer.

Results

(1) Testing basic information

NO.	Multiple alleles	Matching cell lines	Cell bank	EV	Matching instructions
20220301-02	Yes	293T/17 [HEK 293T/17]	DSMZ	1.00	Perfect match

Genotype test results

- Multi allelic genes refer to the phenomenon of genes with three or more alleles.
- The results of this genetic typing are valid.

(2) Description of each sample

- 20220301-02: The DNA typing of this cell line was matched perfectly in the cell line search. The DSMZ database shows that the cell name is 293T/17 [HEK 293T/17], and the cell number corresponds to CRL-11268. Multiple alleles were found in this cell line during this test.

Note: The STR data of the submitted cell line was compared with that of the cell lines listed in ATCC, DSMZ, ExPASy, JCRB, and RIKEN databases. Cell lines that are not included in these databases will not be matched.

(3) Sample typing results

Genotyping results of STR and Amelogenin loci of the cell 20220608-03						
Loci	STR information for submitted sample			STR information for cell bank		
	Name of submitted sample: KGN			Name of cells in cell bank: KGN		
	Allele1	Allele2	Allele3	Allele1	Allele2	Allele3
D5S818	13	13		13	13	
D13S317	9	9		9	9	
D7S820	11	11		11	11	
D16S539	10	12		10	12	
VWA	17	17		17	17	
TH01	9	9		9	9	
AMEL	X	X		X	X	
TPOX	8	9		8	9	
CSF1PO	10	12		10	12	
D12S391	19	21				
FGA	25	26				
D2S1338	25	25				
D21S11	30	32.2				
D18S51	12	14				
D8S1179	13	14				
D3S1358	14	15	17			
D6S1043	13	20				
PENTAE	12	21				
D19S433	13	15.2				
PENTAD	10	13				
D1S1656	15	17				

Other Instructions

(1) Typing scheme and site distribution

	Scheme 1	Scheme 2	Scheme 3	Scheme 4
1	D3S1358	D8S1179	D19S433	AMEL
2	VWA	D21S11	TH01	D1S1656
3	D7S820	D16S539	D13S317	D5S818
4	CSF1PO	D2S1338	TPOX	D12S391
5	PENTAE	PENTAD	D18S51	FGA
6			D6S1043	

Typing scheme and loci

(2) STR database comparison

Our company uses DSMZ tools for cell line comparison, which includes 2455 cell line STR data from ATCC, DSMZ, ExPASy, JCRB, and RIKEN databases. If the cells to be tested are not included in the above cell bank or if they are newly established cell lines, they cannot be compared. Users need to compare their cell typing results with other databases on their own.

(3) References

[1] Authentication testing of HEK 293T and HeLa cell lines have been performed by Shanghai Biowing Applied Biotechnology Co.,Ltd via STR profiling. STR profiles match the standards recommended for HEK 293T and HeLa cell lines authentication.

[2] AGS, NCI-N87, HGC-27 and HEK293 were STR-authenticated on Dec. 8, 2015 by Shanghai Biowing Applied Biotechnology Co. LTD, Shanghai, China.

Experimenter: Jianan Zhang

Reviewer: Ning Qian

Head: Yang Bai

Date of Issue：2022-03-08
